# Novel coumarin-6-sulfonamide-chalcone hybrids as glutathione transferase P1-1 inhibitors

**DOI:** 10.1371/journal.pone.0306124

**Published:** 2024-08-14

**Authors:** Ahmed Sabt, Stefanos Kitsos, Manal S. Ebaid, Veronika Furlan, Panagiota D. Pantiora, Magdalini Tsolka, Eslam B. Elkaeed, Mohamed Farouk Hamissa, Nikolaos Angelis, Ourania E. Tsitsilonis, Anastassios C. Papageorgiou, Urban Bren, Nikolaos E. Labrou

**Affiliations:** 1 Chemistry of Natural Compounds Department, Pharmaceutical and Drug Industries Research Institute, National Research Center, Dokki, Cairo, Egypt; 2 Laboratory of Enzyme Technology, Department of Biotechnology, School of Applied Biology and Biotechnology, Agricultural University of Athens, Athens, Greece; 3 Department of Chemistry, College of Science, Northern Border University, Arar, Saudi Arabia; 4 Faculty of Chemistry and Chemical Engineering, University of Maribor, Maribor, Slovenia; 5 Department of Pharmaceutical Sciences, College of Pharmacy, AlMaarefa University, Diriyah, Saudi Arabia; 6 Institute of Organic Chemistry and Biochemistry, Academy of Sciences, Prague, Czech Republic; 7 Section of Animal and Human Physiology, Department of Biology, National and Kapodistrian University of Athens (NKUA), Athens, Greece; 8 Turku Bioscience Centre, University of Turku and Åbo Akademi University, Turku, Finland; 9 Faculty of Mathematics, Natural Sciences and Information Technologies, University of Primorska, Koper, Slovenia; 10 Institute of Environmental Protection and Sensors, Maribor, Slovenia; Vignan Pharmacy College, INDIA

## Abstract

Multidrug resistance (MDR) mechanisms in cancer cells are greatly influenced by glutathione transferase P1-1 (hGSTP1-1). The use of synthetic or natural compounds as hGSTP1-1 inhibitors is considered an effective approach to overcome MDR. Nine compounds consisting of coumarin-6-sulfonamide linked to chalcone derivatives were synthesized and evaluated for their ability to inhibit hGSTP1-1. Among the synthetic derivatives, compounds **5g**, **5f**, and **5a** displayed the most potent inhibitory effect, with IC_50_ values of 12.2 ± 0.5 μΜ, 12.7 ± 0.7 and 16.3 ± 0.6, respectively. Kinetic inhibition analysis of the most potent molecule, **5g**, showed that it behaves as a mixed-type inhibitor of the target enzyme. An *in vitro* cytotoxicity assessment of **5a**, **5f,** and **5g** against the human prostate cancer cell lines DU-145 and PC3, as well as the breast cancer cell line MCF-7, demonstrated that compound **5g** exhibited the most pronounced cytotoxic effect on all tested cell lines. Molecular docking studies were performed to predict the structural and molecular determinants of **5g**, **5f**, and **5a** binding to hGSTP1-1. In agreement with the experimental data, the results revealed that **5g** exhibited the lowest docking score among the three studied inhibitors as a consequence of shape complementarity, governed by van der Waals, hydrogen bonds and a π-π stacking interaction. These findings suggest that coumarin-chalcone hybrids offer new perspectives for the development of safe and efficient natural product-based sensitizers that can target hGSTP1-1 for anticancer purposes.

## 1. Introduction

Glutathione transferases (GSTs) catalyze the conjugation of glutathione (GSH) to the electrophilic center of xenobiotic compounds [1–4]. GSTs are ubiquitous and multifunctional enzymes that contribute to cell detoxification, metabolism, and apoptosis as well as regulation of cell proliferation and differentiation [1, 2]. Hence, GSTs play a significant role in safeguarding against harmful substances, such as anticancer drugs, pollutants, and carcinogens, by facilitating the nucleophilic attack of reduced glutathione [3, 4]. GSTs are dimeric proteins. Each subunit of the dimer contains a functional region comprising two distinct parts: a binding site for glutathione (G-site) and a binding site for electrophilic substrates (H-site) [5].

Chemotherapy is widely used for treating different types of tumors. However, a significant problem in cancer therapy is the emergence of resistance to chemotherapeutic drugs in cancer cells [6, 7]. Multidrug resistance (MDR) is defined as the ability of cancer cells to withstand the effects of various chemotherapeutic agents [8, 9]. The mechanisms underlying drug resistance include changes in drug transport, leading to reduced entry or increased efflux of drugs from tumor cells and increased expression of GSTs, thus enhancing the conjugation of chemotherapeutic agents, especialy of the alkylating agents (e.g. cisplatin, chlorambucil, melphalan, carmustine, cyclophosphamide, thiotepa) that have been used for the treatment of a wide variety of MDR cancers including multiple myeloma, lymphoma, glioma, prostate, ovarian, bladder, lung, etc [8, 10–13]. Overexpression of GSTs has been noted in a range of cancer types, such as prostate cancer, gastric carcinoma, and acute and chronic lymphoblastic leukemia [14–16]. In particular, the isoenzyme hGSTP1-1 plays an important role in multidrug resistance by increasing the GSH-conjugation of alkylating chemotherapeutic drugs and exercising a regulatory function in the mitogen-activated protein (MAP) kinase pathway. This pathway plays important roles in cellular survival and death signals via protein-protein interactions involving c-Jun N-terminal kinase 1 (JNK1) and apoptosis signal-regulating kinase (ASK1) [2, 7, 17, 18]. Moreover, recent research has revealed that the chaperone function of hGSTP1-1 plays a crucial role in modulating the activity of diverse intracellular proteins [19].

Because of its ability to promote tumor cell resistance in two ways, hGSTP1-1 is an important target for the creation of new compounds designed to combat MDR. This can be accomplished by either suppressing the catalytic function of GSTP1-1 or interfering with its interaction with stress signalling kinases [20]. Additionally, hGSTP1-1 is a desirable target for drug development because it fulfills two crucial requirements: significant association with diseases (target validation) and potential for being targeted by drugs (target tractability) [21–25]. GST inhibitors can either sensitize drug-resistant tumors overexpressing GSTs or can be used as prodrugs activated *in vivo* by GSTs [21–23]. Over the years, numerous powerful inhibitors have been created that bind either to the G-site or the H-site of the GSTs [4, 10, 21, 23]. For example, ethacrynic acid and the glutathione analogues ezatiostat (TER199) and canfosfamide (TER 286) have been clinically studied [8]. Other examples include ethacraplatin, a bifunctional drug composed of a cisplatin molecule conjugated by two ethacrynic acid ligands [26], the benzoxadiazol derivative 6-(7-nitro-2,1,3-benzoxadiazol-4-ylthio)hexanol (NBDHEX), which triggers apoptosis in several cancer cells [27], and auranofin, an antiarthritic gold phosphine compound for which recent studies showed that hGSTP1-1 is one of its targets [28].

**Fig 1:**
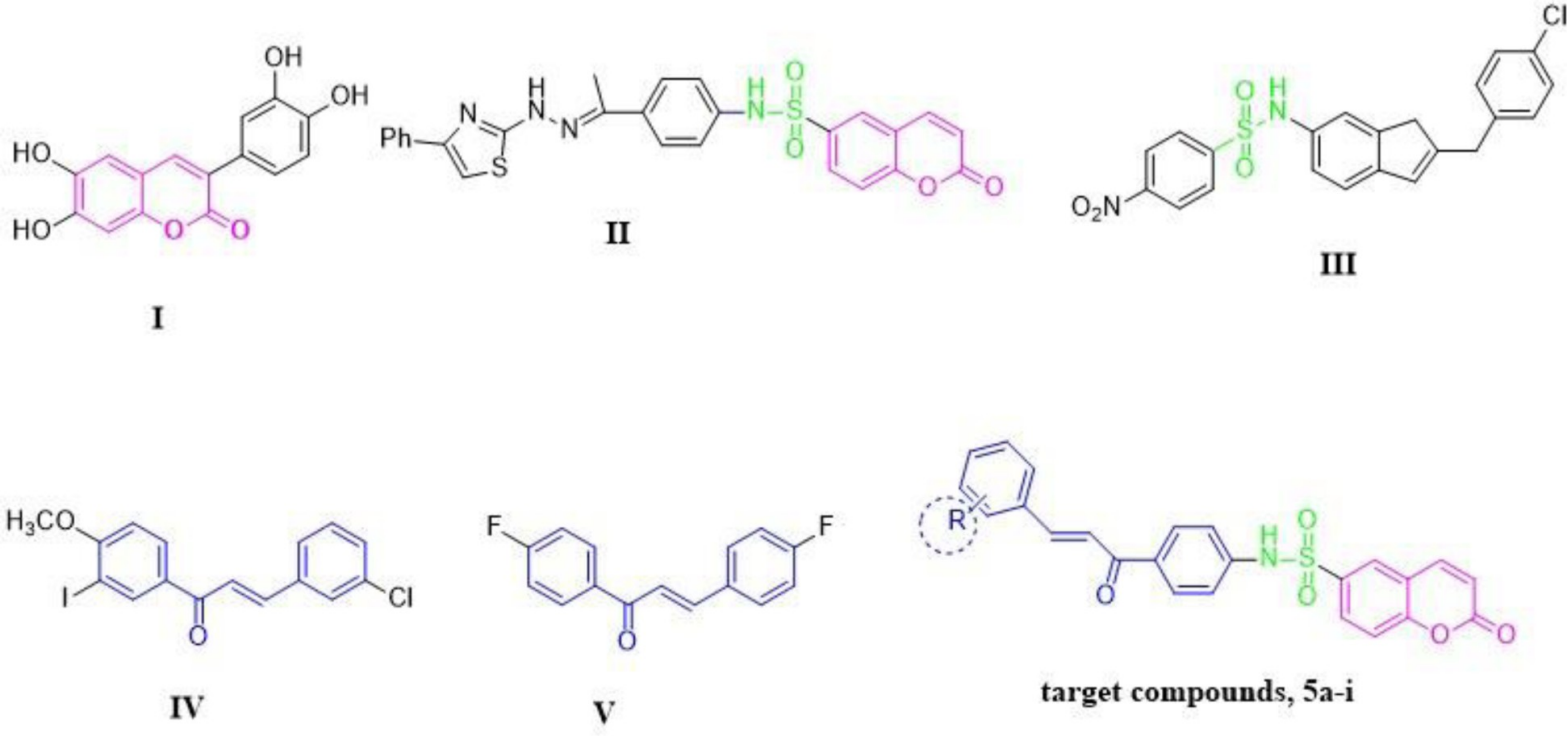
Structures of representative hybrids of bioactive cores I-V and the newly designed compounds 5a-i reported in the present study.

Owing to their varied molecular compositions and significant bioavailability, natural products are employed in the field of medicinal chemistry for the development of novel chemical structures [29]. Recently, much attention has been paid to finding natural chemopreventive substances, especially polyphenolic compounds [30]. A variety of natural products, including coumarins and chalcones, have been shown to inhibit human GSTs *in vitro* [20–34]. Additionally, coumarin derivatives exhibit a variety of biological and pharmacological properties [35] such as anticoagulant, anti-inflammatory, antiviral, antimicrobial, antileishmanial, and anticancer [36–43]. Although there is limited research on the effects of coumarins on human GST activity, arylcoumarin **I** (Fig 1) showed significant GST inhibition [44, 45]. In 2018, Sabt *et al*. developed a new series of coumarin-6-sulfonamides with anticancer activity [40]. Among them, compound **II** (Fig 1) was the most active with IC_50_ = 3.48 ± 0.28 μM towards HepG2 cells, 1.5 times higher than doxorubicin (IC_50_ = 5.43 ± 0.24 μM). On the other hand, sulfonamide derivatives are pluripotent compounds that have shown promising biological activities, such as anti-carbonic anhydrase and anticancer properties [21, 46–49]. Additionally, a variety of compounds containing sulfonamido groups can be accommodated at the H-site of GST and act as GST inhibitors or substrates [21, 47]. For instance, compound **III** (Fig 1) showed potency similar to that of ethacrynic acid, the reference drug, as the most active hGSTP1-1 inhibitor with a value of IC_50_ = 10.2μM [50]. Both natural and synthetic chalcones are reported to have a significant potential as drugs with numerous biological activities [51, 52]. Chalcones have been found to disrupt the cell cycle and induce apoptosis. Moreover, they can inhibit tubulin polymerization and target specific kinases that are crucial for the proliferation and survival of cancer cells such as compound **IV** (Fig 1) [52]. Özaslan *et al*. developed several chalcone compounds that target GSTs, and compound **V** (Fig 1) was the most active derivative [32].

In recent years, the development of drugs in pharmaceutical and medicinal chemistry has been aided by the use of molecular hybridization. This approach involves the combination of different scaffolds to create novel analogs with enhanced biological activities [53].

The present work aimed to investigate whether coumarin-6-sulfonamide-chalcone hybrids can inhibit hGSTP1-1. For this purpose, a series of derivatives containing a coumarin core with substituted chalcone moieties was synthesized. Following their synthesis, the compounds were evaluated for their inhibitory potency against hGSTP1-1. The most effective compounds were further evaluated for their cytotoxicity against three different cancer cell lines. Additionally, molecular docking was conducted to predict the structural and molecular determinants of the interactions between the most biologically active derivatives and the target enzyme hGSTP1-1. Overall, this study provides new insights into the design of hybrid natural products for the creation of effective and safe chemical sensitizers that specifically target hGSTP1-1.

## 2. Results and discussion

### 2.1. Synthesis and characterization of coumarin derivatives

**Scheme 1:**
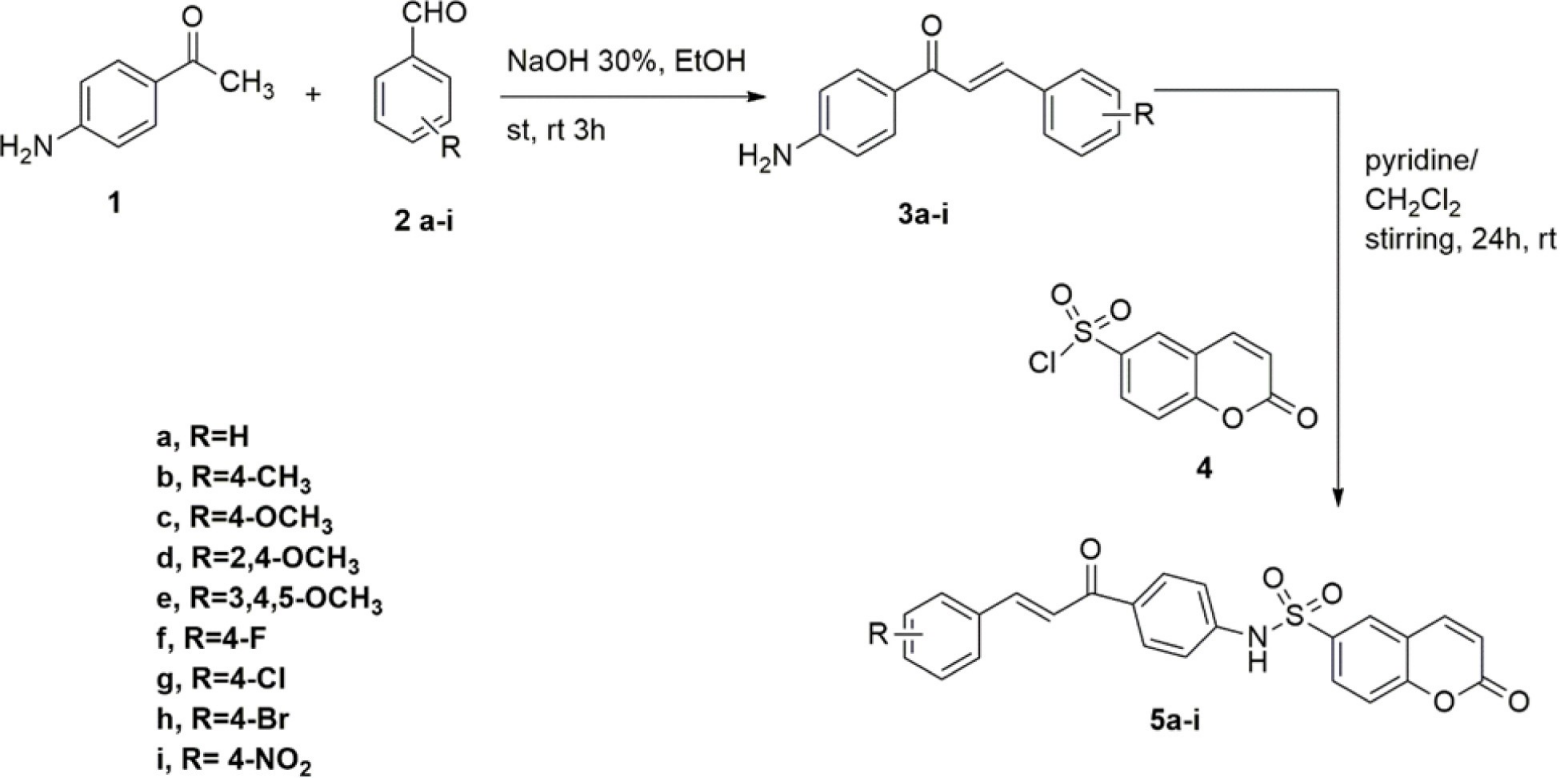


The synthesis of the coumarin-6-sulfonamide-chalcone conjugates is shown in **Scheme 1**. The 4-aminochalcone building blocks **3a-i** were synthesized using the Claisen-Schmidt condensation of 4-aminoacetophenones and aryl aldehydes substituted with electron-withdrawing or electron-donating groups, as previously reported [54]. The reaction of coumarin-6-sulfonylchloride with 4-aminochalcone derivatives **(3a-i)** in methylene chloride in the presence of 1 mL pyridine at room temperature afforded the target coumarin-6-sulfonamide bearing chalcone derivatives **(5a-i)**. ^1^H NMR and ^13^C NMR spectral data were used to determine the structures of the derivatives **5a-i**. The ^1^H NMR spectrum showed characteristic signals ranging from 7.60 to 8.08 ppm, corresponding to the hydrogens of the olefinic double bond. The observed signals manifested as doublets, occurring in pairs, with coupling constants spanning from 15.6 to 16.0 Hz, thereby suggesting a trans configuration. In the ^13^C NMR spectrum, the most deshielded signals, exhibiting chemical shifts in the range 187–189 ppm, were attributed to the carbonyl group of the trans-enone bridge.

### 2.2. Enzyme inhibition analysis

By conducting enzyme activity assays, the effectiveness of the coumarin derivatives in inhibiting hGSTP1-1 was assessed at a concentration of 10 μM. As shown in Table 1, all the synthesized compounds demonstrated inhibitory activities, except for compound **5e**, which exhibited no activity. Compounds **5i, 5c, 5d, 5h,** and **5b** exhibited medium inhibition potency (45.4–71.1%), whereas compounds **5a, 5f,** and **5g** showed the highest potency (>85%) and were chosen for additional examination.

**Table 1:** Assessment of hGSTP1-1 isoenzyme inhibition by coumarin derivatives. Enzyme assays were performed in triplicate, and enzyme inhibition values are expressed as the mean ± SE.

Compound	Enzyme Inhibition (%)
**5a**	84.6 ± 0.1
**5b**	62.8 ± 0.1
**5c**	67.0 ± 0.0
**5d**	59.3 ± 0.1
**5e**	*
**5f**	86.9 ± 0.0
**5g**	87.5 ± 0.0
**5h**	71.1 ± 0.1
**5i**	45.4 ± 0.1

* Coumarin derivative **5e** did not exhibit significant inhibition.

**Fig 2:**
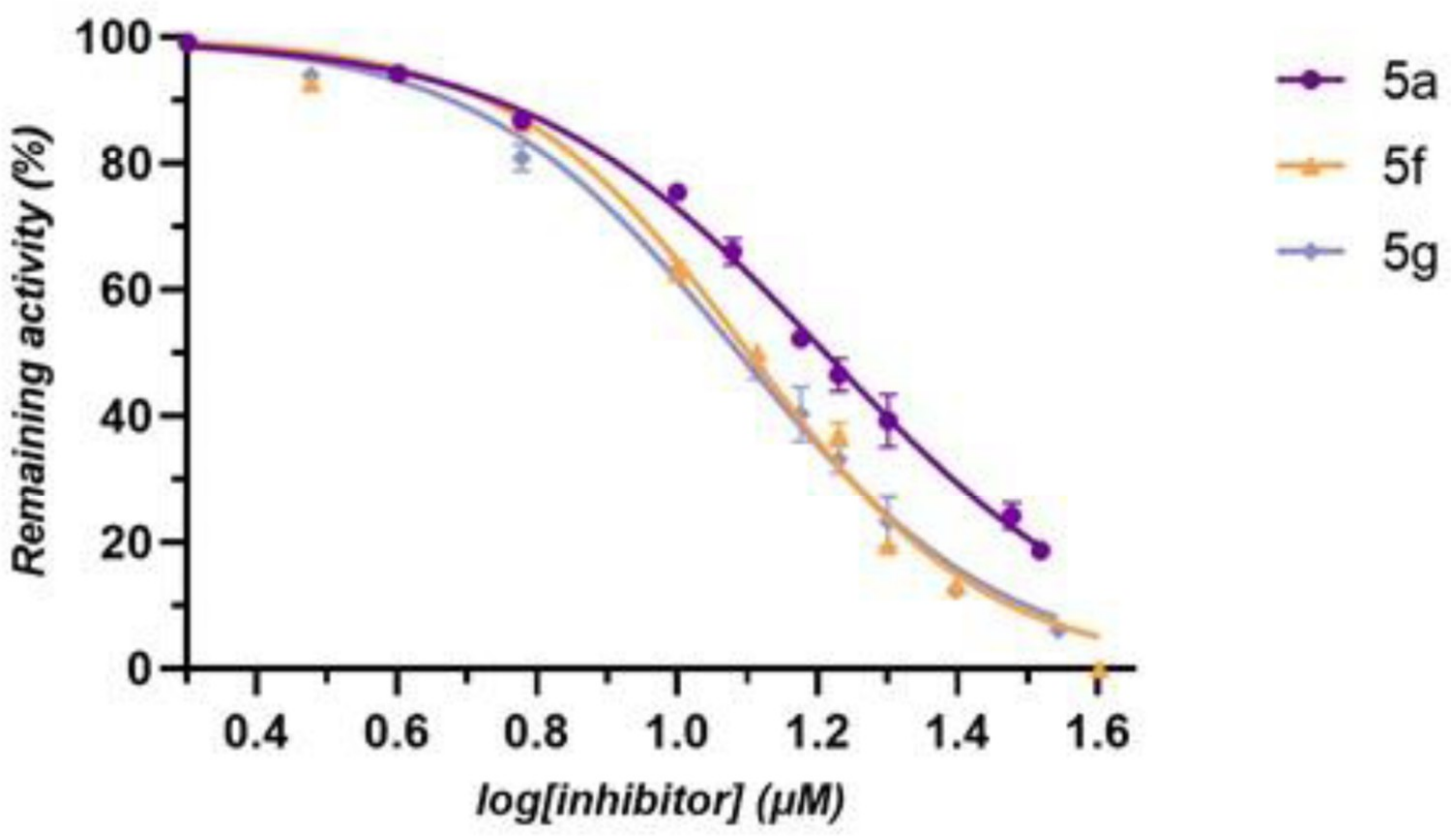
Concentration-response inhibition curves for the determination of IC_50_ values of the most potent inhibitors 5a, 5f, and 5g against hGSTP1-1. Data are reported as the mean ± standard error of three replicates.

Compounds that exerted the highest inhibitory activity were selected for IC_50_ determination using dose inhibition studies. A schematic representation of the results is shown in Fig 2 and a summary of the corresponding IC_50_ values is presented in **Table 2**. Coumarin derivatives **5g** and **5f** showed a significant effect on enzyme activity with similar ΙC_50_ values of 12.2 ± 0.5 μΜ and 12.7 ± 0.7 μΜ, respectively. On the other hand, compound **5a** appeared to be a moderate inhibitor with an IC_50_ value of 16.28 ± 0.56 μΜ.

**Table 2:** IC_50_ values of compounds 5a, 5f, and 5g towards hGSTP1-1 obtained by concentration-response inhibition studies. All experiments were performed in triplicate, and IC_50_ values are expressed as the mean ± SE.

Compound	IC_50_ (μΜ)
**5a**	16.3 ± 0.6 μΜ
**5f**	12.7 ± 0.7 μΜ
**5g**	12.2 ± 0.5 μΜ

Several recent studies have reported the synthesis of diverse synthetic compounds that exhibit varying levels of inhibitory potency towards hGSTP1-1 or hGSTA1-1, such as benzoxadiazoles [55, 56], dihydroxybenzophenones [57], pyrroles [58], and calix [4] arenes [59]. Additionally, numerous natural products have been reported to act as potent hGSTP1-1 inhibitors [34, 60–62]. Recently, Ozalp *et al*. [63], reported a series of arylcoumarin and biscoumarin derivatives as GSTP1-1 inhibitors.

**Fig 3:**
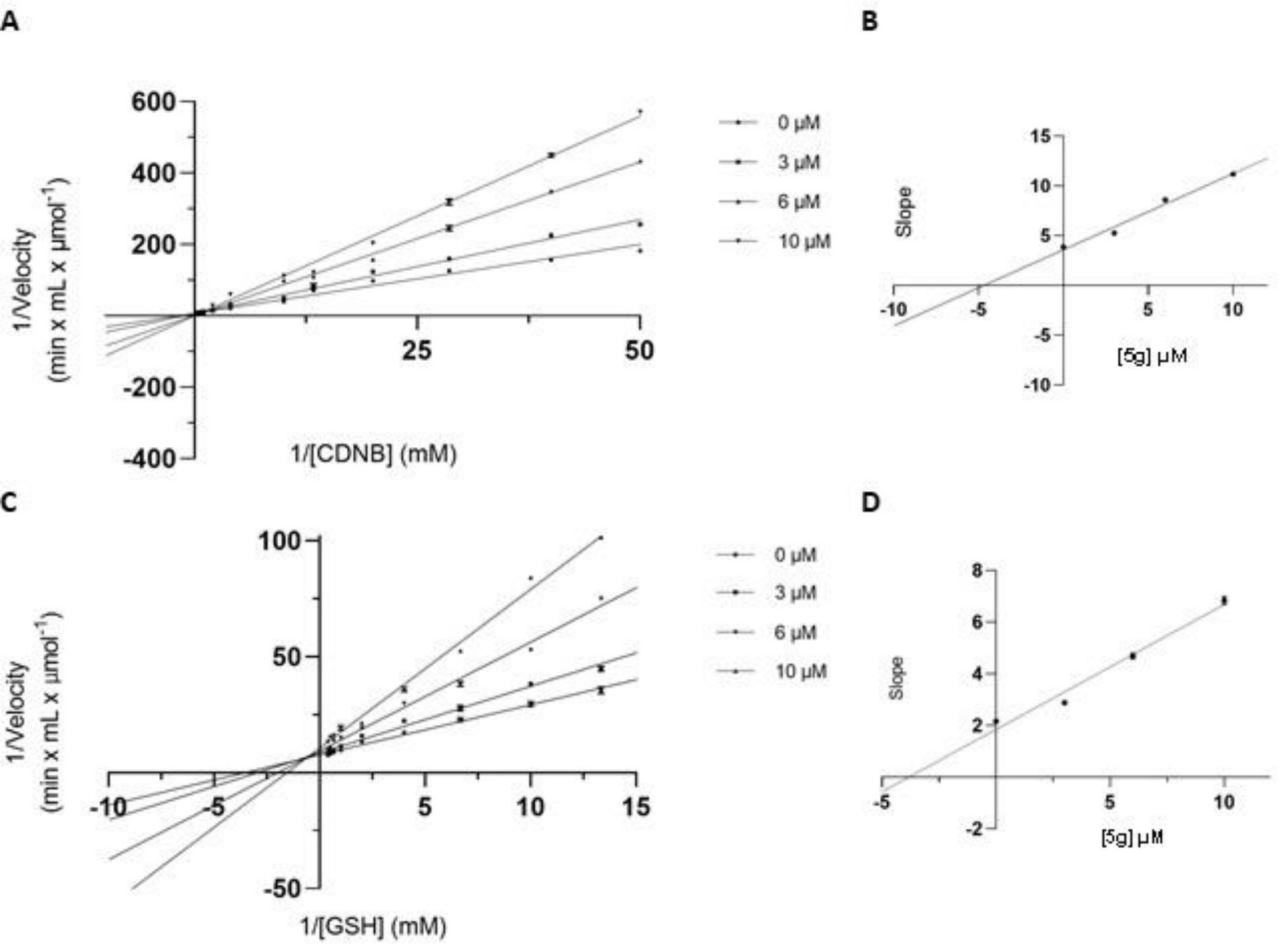
Kinetics inhibition studies. (A) Lineweaver–Burk plot of the inhibition of hGSTP1-1 isoenzyme using CDNB as a variable substrate (20–3,000 μΜ) at different constant concentrations of **5d**. (B) Secondary plot of the slopes of each Lineweaver–Burk line as a function of **5g** concentration. (C) Lineweaver–Burk plot of the inhibition of hGSTP1-1 isoenzyme using GSH as a variable substrate (75–2,500 μΜ) at different constant concentrations of **5g**. (D) Secondary plot of the slopes of each Lineweaver–Burk line as a function of **5g** concentration.

Kinetic inhibition studies were performed using the strongest inhibitor **(5g)** to determine both the binding mode to hGSTP1-1 and the type of observed inhibition. In the presence of variable concentrations of 1-chloro-2,4-dinitrobenzene (CDNB), **5g** behaves as a non-competitive, purely mixed-type inhibitor, as indicated by the linearity of the double reciprocals (Lineweaver-Burk) graph and the derived secondary plot (**Fig 3A and 3B**). These results suggest that **5g** has the ability to bind to both the free hGSTP1-1, with an inhibition constant K_i_ of 4.69 ± 0.11 μΜ, and the hGSTP1-1–CDNB complex, with an inhibition constant K_i_΄ of 19.25 ± 1.20 μΜ. Similarly, the same inhibitory behavior was observed when GSH was used as the variable substrate (**Fig 3C and 3D**), with calculated inhibition constants K_i_ = 3.82 ± 0.16 μΜ and K_i_΄ = 25.39 ± 0.60 μΜ. The large observed differences between the inhibition constants K_i_ and K_i_΄ (about 4-5-times) clearly indicates that **5g** has a tendency to bind with higher affinity with the free enzyme rather with the hGSTP1-1–CDNB or hGSTP1-1–GSH complexes. Similarly, non-competitive/mixed-type inhibition has also been observed with other synthetic or natural product inhibitors of hGSTP1-1 or other evolutionarily distant GSTs [34, 55–61]. This common kinetic behavior suggests an evolutionarily conserved inhibition mechanism for all the GSTs.

### 2.3. Evaluation of the cytotoxic effects of coumarin derivatives on DU-145, PC3, and MCF-7 cell lines using *in vitro* methods

**Fig 4:**
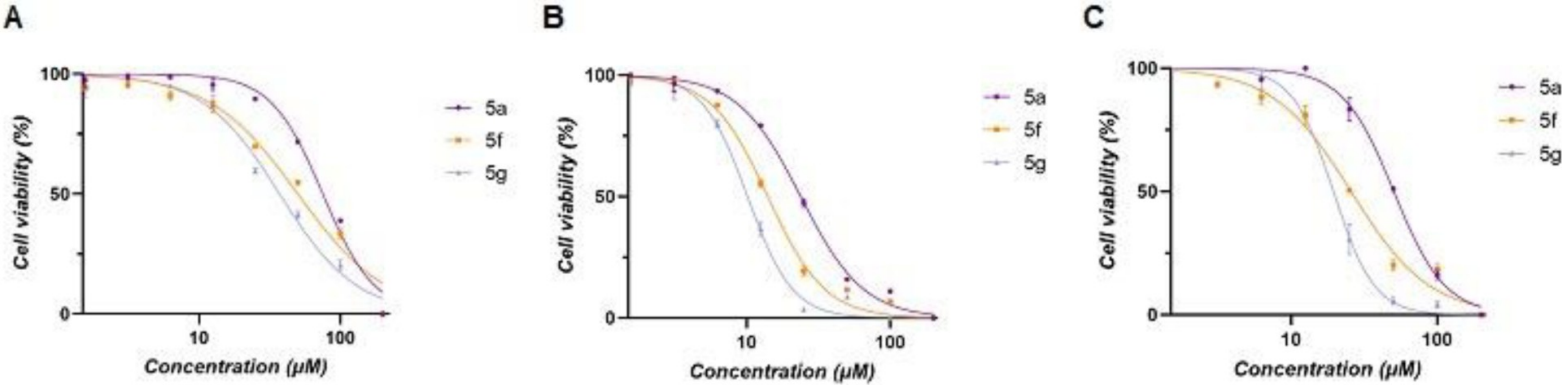
Dose-dependent cytotoxicity effect of the inhibitors 5a, 5f, and 5g against three human tumor cell lines. (A): DU-145; (B): PC3; (C) MCF-7. The data were analyzed using GraphPad Prism 9.3.1.

Cytotoxic evaluations were conducted on the most efficacious inhibitors targeting three representative cancer cell lines, namely DU-145, PC3, and MCF-7. The cytotoxicity of these inhibitors was assessed against two prostate cancer cell lines, PC3 and DU-145, and one breast cancer cell line, MCF-7. Cell viability was determined using the standard MTT protocol, and the half-maximal cytotoxic concentration (CC_50_) values were calculated for the three most potent inhibitors **5a, 5f,** and **5g**. The results are summarized in **Table 3** and the dose-dependent cytotoxicity effect of **5a, 5f,** and **5g** is illustrated in **Fig 4A–4C**. The results indicate an excellent correlation between the CC_50_ **(Table 3)** and IC_50_ values determined by enzyme kinetics analysis **(Table 2)**. The most potent inhibitor of hGSTP1-1, **5g**, showed the greatest cytotoxicity against the three cancer cell lines. In contrast, DU-145 appeared to be the least sensitive of the three coumarin derivatives, with both PC3 and MCF-7 being the most sensitive. The cytotoxic effect of **5g** on MCF-7 cells was much greater than that of other natural substances, such as catechin and gossypol [25].

**Table 3:** Half-maximal cytotoxic concentration (CC_50_) values for the most potent inhibitors 5a, 5f, and 5g against DU-145, PC3, and MCF-7 cell lines. All experiments were performed in triplicate, and the CC_50_ values were expressed as the mean ± SE.

Compound	CC_50_ against DU-145 (μΜ)	CC_50_ against PC3 (μΜ)	CC_50_ against MCF-7 (μΜ)
5a	71.4 ± 5.4	25.3 ± 2.2	46.8 ± 5.1
5f	47.3 ± 2.7	14.7 ± 0.9	24.7 ± 1.5
5g	36.6 ± 2.7	11.9 ± 2.5	17.4 ± 2.9

### 2.4. Molecular docking studies

#### 2.4.1. Molecular docking of 5a, 5f, and 5g to hGSTP1-1

Molecular docking was performed using the CANDOCK algorithm and the RMR6 scoring function to investigate the binding mechanisms of coumarin derivatives **5a, 5f,** and **5g** in the active pocket of the target enzyme hGSTP1-1 [64]. Following an initial visual inspection, the binding patterns of **5g, 5f**, and **5a** at the active site of hGSTP1-1 were selected based on their lowest docking score values, as shown in **Table 4**.

**Table 4:** The lowest docking score values of the studied hGSTP1-1–coumarin derivative complexes.

Coumarin derivative	Docking score values (arbitrary units)
**5a**	-36.84
**5f**	-42.18
**5g**	-45.97

As indicated in **Table 4**, all examined coumarin derivatives exhibited a strong affinity for binding to hGSTP1-1, with docking score values below -30 arbitrary units. The findings from the molecular docking analysis align with the experimental data obtained, suggesting that the investigated coumarin derivatives possess inhibitory properties against the target enzyme hGSTP1-1. Additionally, the molecular docking findings revealed that the coumarin derivative 5g exhibited a higher affinity for binding to hGSTP1-1 than compounds **5a** and **5f** as indicated by the lowest docking score value (-45.97 arbitrary units). As a result, **5g** was selected for further analysis.

#### 2.4.2. Binding mode of 5a, 5f, and 5g at the active site of hGSTP1-1

**Fig 5:**
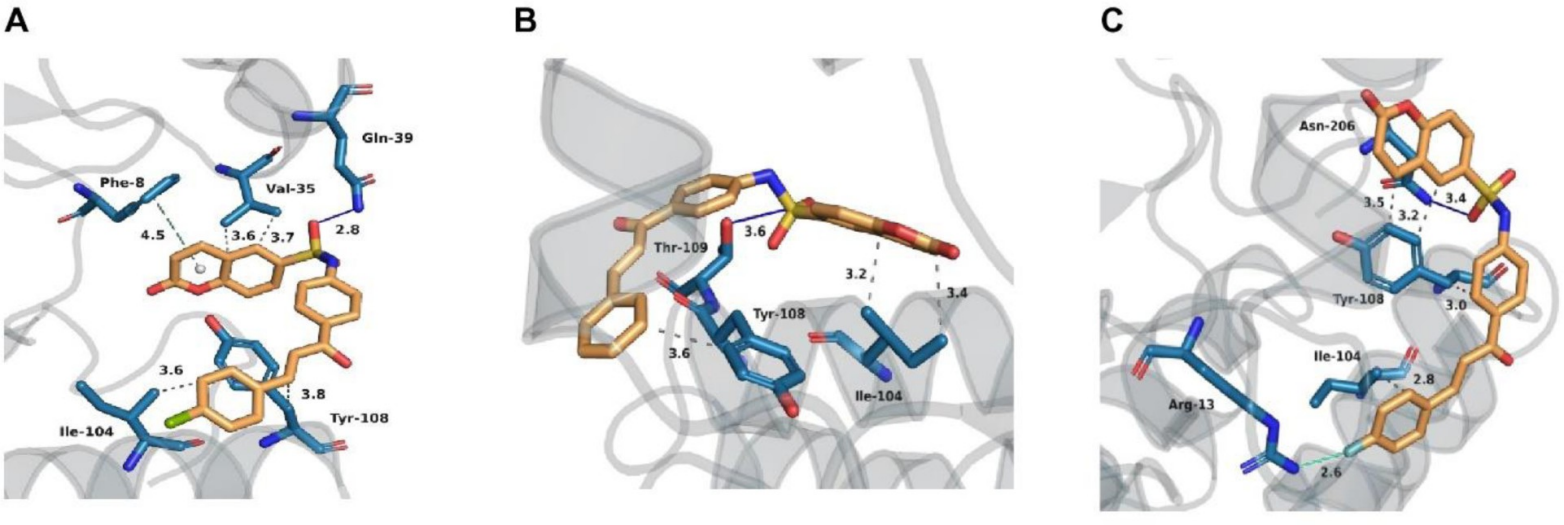
The predicted interaction of interaction of 5g, 5a, and 5f with hGSTP1-1. (A) Binding mode of compound **5g** at the active site of hGSTP1-1 with the lowest docking score value. (B) Binding mode of compound **5a** at the active site of hGSTP1-1 with the lowest docking score value. (C) Binding mode of compound **5f** at the active site of hGSTP1-1 with the lowest docking score value. In subfigures a), b), and c) carbon atoms of compounds **5g**, **5a**, and **5f** are depicted in orange, while carbon atoms of hGSTP1-1 amino-acid residues are presented in light blue color. The oxygen atoms are red, nitrogen atoms are dark blue, sulphur atom is yellow, chlorine atom is green, and fluorine atom is light green color. Van der Waals interactions are presented with gray dashed lines, hydrogen bonds are depicted with dark blue lines, π-π stacking interaction is presented with green dashed line, and halogen interaction with cyan line. Hydrogen atoms are omitted for reasons of clarity.

The detailed interactions of **5a**, **5f**, and **5g** with hGSTP1-1 were assessed with the Protein Ligand Interaction Profiler (PLIP) [65] and the results are presented in Fig 5.

From the binding mode shown in **Fig 5**, it can be observed that coumarin derivative **5g** forms nonpolar van der Waals interactions interactions with amino acid residues Ile104, Val35, and Tyr108 at distances 3.6, 3.65, 3.7 and 3.8 Å, respectively. **5g** is additionally stabilized through a hydrogen bond with nearby residue Gln39 at a distance of 2.8 Å as well as by a π-π stacking interaction with residue Phe8 at a distance of 4.5 Å. The hydrophobic interactions from G-site are, therefore, crucial for stabilizing the **5g** inhibitor at the active site of hGSTP1-1, while hydrogen bonds play a minor role. Furthermore, the interactions of **5a** and **5f** with the binding site amino-acid residues are shown in Fig 5B and 5C, respectively. From these two figures, it can be observed that similar amino acid residues from the G-site play an important role in the binding of the coumarin derivatives to hGSTP1-1, confirming that all three derivatives (**5g**, **5f**, and **5a**) occupy the same site.

## 3. Conclusion

Over the past two decades, extensive studies have shed light on the role of GSTs on therapeutic response to chemotherapy. Studies have shown that GSTs play a significant role in chemotherapy through their function to deactivate anticancer drugs and/or control cell signaling pathways. In this study, we report the synthesis of novel hybrid compounds based on a coumarin-6-sulfonamide scaffold and investigate their inhibitory and cytotoxic potency against hGSTP1-1 and three cancer cell lines (DU-145, PC3, and MCF-7). The results revealed that **5g** displayed the highest inhibition and cytotoxicity potency and behaved as a mixed-type inhibitor of hGSTP1-1. It is suggested that **5g** may have a synergistic effect on the suppression (cytotoxicity) and chemosensitization (hGSTP1-1 inhibition) of cancer cells. Molecular docking indicated that hydrogen bonds, a π–π stacking interaction, and nonpolar van der Waals interactions play a crucial role in the binding and inhibitory activities of **5g.** Overall, our data provide new insights into the development of natural product-based hybrid molecules that are potent inhibitors towards hGSTP1-1 and function as effective cancer chemosensitizers.

## 4. Experimental section

### 4.1. Chemistry

Melting points were determined using an Electrothermal IA 9000 apparatus and were uncorrected. Elemental analyses were performed at the Micro-Analytical Central Services Laboratory, Faculty of Science, Cairo University, Egypt. ^1^H-NMR and ^13^C-NMR spectra were measured using Bruker Avance II™ 400 MHz spectrometers (Bruker Biospin AG, Fällanden, Switzerland) in Prague, Czech Republic. The reactions were followed by TLC (silica gel, aluminium sheets 60 F254, Merck) using chloroform/methanol (9.5:0.5 v/v) as eluent and sprayed with iodine-potassium. Compounds **3a-i** were previously prepared [66, 67]. The characterization data of all the newly synthesized compounds are reported below.

#### 4.1.1. General procedure for the preparation of coumarin-6-sulfonamide chalcones 5a-i

As a catalytic reaction, a mixture of coumarin-6-sulfonylchloride **(4)** (0.001 mol) and 4-aminochalcone derivatives (0.001 mol) **3a-i** in methylene chloride and 1mL pyridine were stirred at room temperature for 24 hours. The reaction mixture was then put onto ice and acidified with diluted HCl to produce a precipitate, which was then filtered, dried, and crystalized with ethanol to get the target compounds **5a-i**.

N-(4-cinnamoylphenyl)-2-oxo-2*H*-chromene-6-sulfonamide **(5a).**

Yellow powder; yield 81%, m.p. 128–129°C. ^1^H NMR (400 MHz, DMSO-d_6_): δ = 6.60 (d, 1H, *J* = 9.6 Hz, H3 of coumarin), 7.28 (d, 2H, *J* = 8.8 Hz, H-Ar), 7.43–7.44 (m, 3H, H-Ar), 7.57(d, 1H, *J* = 8.8 Hz, H-Ar), 7.66 (d, 1H, *J* = 15.6 Hz, CH =), 7.83–7.86 (m, 3H, CH = and 2H-Ar), 7.96 (dd, 1H, *J* = 2.4 and 8.8 Hz, H-Ar), 8.06 (d, 2H, *J* = 8.8 Hz, H-Ar), 8.18 (d, 1H, *J* = 9.6 Hz, H4 of coumarin), 8.33 (d, 1H, *J* = 2.4 Hz, H5 of coumarin), 11.06 (s, 1H, NH), ^13^C NMR (101 MHz, DMSO-d_6_): δ = 113.43(C3 of Coumarin), 118.40, 118.45, 118.68, 119.53, 122.27, 128.31, 128.94, 129.26, 129.36, 130.00, 130.73, 131.02, 131.59, 133.24, 135.12, 135.61, 142.43, 143.79, 144.05, 156.56(C9 of Coumarin), 159.50(C2 of Coumarin), 188.18(C = O). Anal. Calcd for C_24_H_17_NO_5_S (431.46) C, 66.81; H, 3.97; N, 3.25; Found: C, 66.99; H, 4.14; N, 3.40.

2-oxo-N-(4-(3-(p-tolyl)acryloyl)phenyl)-2*H*-chromene-6-sulfonamide **(5b)**

Yellow powder; yield 77%, m.p. 202–204°C. ^1^H NMR (400 MHz, DMSO-d_6_): δ = 2.46 (s, 3H, CH_3_), 6.59 (d, 1H, *J* = 9.6 Hz, H3 of coumarin), 6.98 (d, 2H, *J* = 8.8 Hz, H-Ar), 7.26 (d, 2H, *J* = 8.8 Hz, H-Ar), 7.56 (d, 1H, *J* = 8.8 Hz, H-Ar), 7.62 (d, 1H, *J* = 15.6 Hz, CH =), 7.68 (d, 1H, *J* = 15.6 Hz, = CH), 7.78 (d, 2H, *J* = 8.8 Hz, H-Ar), 7.97 (dd, 1H, *J* = 2.4 and 8.8 Hz, H-Ar), 8.03 (d, 2H, *J* = 8.8 Hz, H-Ar), 8.17 (d, 1H, *J* = 9.6 Hz, H4 of coumarin), 8.32 (d, 1H, *J* = 2.4 Hz, H5 of coumarin), 11.01 (s, 1H, NH), ^13^C NMR (101 MHz, DMSO-d_6_): δ = 26.87(CH_3_), 114.85, 118.39, 118.43, 118.57, 118.70, 119.52, 119.72, 124.67, 127.76, 128.29, 129.99, 130.57, 131.16, 133.54, 135.62, 142.20, 143.78, 144.17, 149.46, 156.86 (C9 of Coumarin), 159.50 (C2 of Coumarin), 188.18(C = O). Anal. Calcd for C_25_H_19_NO_5_S (445.10) C, 67.40; H, 4.30; N, 3.14; Found: C, 67.58; H, 4.47; N, 3.33.

N-(4-(3-(4-methoxyphenyl)acryloyl)phenyl)-2-oxo-2*H*-chromene-6-sulfonamide **(5c)**

Yellow powder; yield 49%, m.p. 238–239°C. ^1^H NMR (400 MHz, DMSO-d_6_): δ = 3.80 (s, 3H, OCH_3_), 6.59 (d, 1H, *J* = 9.6 Hz, H3 of coumarin), 6.98 (d, 2H, *J* = 8.8 Hz, H-Ar), 7.27 (d, 2H, *J* = 8.8 Hz, H-Ar), 7.57 (d, 1H, *J* = 8.8 Hz, H-Ar), 7.63 (d, 1H, *J* = 15.6 Hz, CH =), 7.69 (d, 1H, *J* = 15.6 Hz, = CH), 7.78 (d, 2H, *J* = 8.8 Hz, H-Ar), 7.98 (dd, 1H, *J* = 2.4 and 8.8 Hz, H-Ar), 8.03 (d, 2H, *J* = 8.8 Hz, H-Ar), 8.17 (d, 1H, *J* = 9.6 Hz, H4 of coumarin), 8.33 (d, 1H, *J* = 2.4 Hz, H5 of coumarin), 11.02 (s, 1H, NH), ^13^C NMR (101 MHz, DMSO-d_6_): δ = 55.83(OCH_3_), 114.85, 118.38, 118.43, 118.71, 119.52, 119.71, 127.75, 128.29, 130.00, 130.57, 131.16, 133.55, 135.63, 142.21, 143.78, 144.05, 156.50 (C9 of Coumarin), 159.50 (C2 of Coumarin), 161.77(OCH_3_-C), 187.88(C = O). Anal. Calcd for C_25_H_19_NO_6_S (461.09) C, 65.07; H, 4.15; N, 3.04; Found: C, 65.24; H, 4.34; N, 3.19.

N-(4-(3-(2,4-dimethoxyphenyl)acryloyl)phenyl)-2-oxo-2*H*-chromene-6-sulfonamide **(5d)**

Yellow powder; yield 71%, m.p. 208–210°C. ^1^H NMR (400 MHz, DMSO-d_6_): δ = 3.83 (s, 3H, OCH_3_), 3.88 (s, 3H, OCH_3_), 6.60–6.63 (m, 3H, H3 of coumarin and 2H-Ar), 7.26 (d, 2H, *J* = 8.8 Hz, H-Ar), 7.57 (d, 1H, *J* = 8.8 Hz, H-Ar), 7.65 (d, 1H, *J* = 15.6 Hz, CH =), 7.84 (d, 1H, *J* = 8.4 Hz, H-Ar), 7.90 (d, 1H, *J* = 15.6 Hz, = CH), 7.97–8.01 (m, 3H, H-Ar), 8.17 (d, 1H, *J* = 10.0 Hz, H4 of coumarin), 8.32 (d, 1H, *J* = 2.0 Hz, H5 of coumarin), 11.00 (s, 1H, NH), ^13^C NMR (101 MHz, DMSO-d_6_): δ = 56.00 (OCH_3_), 56.28(OCH_3_), 98.74, 106.79, 116.35, 118.39, 118.43, 118.76, 119.22, 119.52, 128.28, 130.00, 130.44, 130.55, 133.75, 135.64, 138.84, 142.07, 143.80, 156.50 (C9 of Coumarin), 159.51(C2 of Coumarin), 160.39 (OCH_3_-C), 163.52 (OCH_3_-C), 187.99(C = O). Anal. Calcd for C_26_H_21_NO_7_S (491.10) C, 63.54; H, 4.31; N, 2.85; Found: C, 63.71; H, 4.49; N, 3.00.

2-oxo-N-(4-(3-(3,4,5-trimethoxyphenyl)acryloyl)phenyl)-2*H*-chromene-6-sulfonamide **(5e)**

Yellow powder; yield 55%, m.p. 235–237°C. ^1^H NMR (400 MHz, DMSO-d_6_): δ = 3.70 (s, 3H, OCH_3_), 3.84 (s, 6H, 2OCH_3_), 6.60 (d, 1H, *J* = 9.6 Hz, H3 of coumarin), 7.18 (s, 2H, 2H-Ar), 7.27 (d, 2H, *J* = 8.8 Hz, H-Ar), 7.57 (d, 1H, *J* = 8.8 Hz, H-Ar), 7.61 (d, 1H, *J* = 15.6 Hz, CH =), 7.77 (d, 1H, *J* = 15.2 Hz, = CH), 7.98 (dd, 1H, *J* = 2.4 and 8.8 Hz, H-Ar), 8.05 (d, 2H, *J* = 8.8 Hz, H-Ar), 8.17 (d, 1H, *J* = 9.6 Hz, H4 of coumarin), 8.32 (d, 1H, *J* = 2.0 Hz, H5 of coumarin), 11.04 (s, 1H, NH), ^13^C NMR (101 MHz, DMSO-d_6_): δ = 56.56 (2OCH_3_), 60.59 (OCH_3_), 106.91, 118.41, 118.45, 118.64, 119.53, 121.41, 128.28, 129.99, 130.67, 130.71, 133.33, 135.64, 140.13, 142.39, 143.78, 144.52, 153.53 (OCH_3_-2C), 156.51(C9 of Coumarin), 159.49 (C2 of Coumarin), 187.90 (C = O). Anal. Calcd for C_27_H_23_NO_8_S (521.11) C, 62.18; H, 4.45; N, 2.69; Found: C, 62.36; H, 4.63; N, 2.85.

N-(4-(3-(4-fluorophenyl)acryloyl)phenyl)-2-oxo-2*H*-chromene-6-sulfonamide **(5f)**

Yellow powder; yield 62%, m.p. 224–226°C. ^1^H NMR (400 MHz, DMSO-d_6_): δ = 6.60 (d, 1H, *J* = 9.6 Hz, H3 of coumarin), 7.23–7.30 (m, 4H, H-Ar), 7.57 (d, 1H, *J* = 8.8 Hz, H-Ar), 7.65 (d, 1H, *J* = 15.6 Hz, CH =), 7.79 (d, 1H, *J* = 15.6 Hz, = CH), 7.90–7.93 (m, 2H, H-Ar), 7.98 (dd, 1H, *J* = 2.4 and 8.8 Hz, H-Ar), 8.05 (d, 2H, *J* = 8.8 Hz, H-Ar), 8.17 (d, 1H, *J* = 9.6 Hz, H4 of coumarin), 8.33 (d, 1H, *J* = 2.0 Hz, H5 of coumarin), 11.05 (s, 1H, NH), ^13^C NMR (101 MHz, DMSO-d_6_): δ = 116.26, 116.48, 118.39, 118.44, 118.57, 118.66, 119.52, 122.16, 128.31, 130.00, 130.32, 130.73, 131.56, 131.65, 131.83, 133.22, 135.61, 142.44, 142.80, 143.78, 156.51(C9 of Coumarin), 159.50 (C2 of Coumarin), 162.58 (C-F), 187.92 (C = O). Anal. Calcd for C_24_H_16_FNO_5_S (449.07) C, 64.14; H, 3.59; N, 3.12; Found: C, 64.32; H, 3.78; N, 3.30.

N-(4-(3-(4-chlorophenyl)acryloyl)phenyl)-2-oxo-2*H*-chromene-6-sulfonamide **(5g).**

Pale yellow powder; yield 59%, m.p. 254–255°C. ^1^H NMR (400 MHz, DMSO-d_6_): δ = 6.60 (d, 1H, *J* = 9.6 Hz, H3 of coumarin), 7.27 (d, 2H, *J* = 8.8 Hz, H-Ar), 7.50 (d, 2H, *J* = 8.4 Hz, H-Ar), 7.57 (d, 1H, *J* = 8.8 Hz, H-Ar), 7.64 (d, 1H, *J* = 15.6 Hz, CH =), 7.85–7.89 (m, 3H, = CH and 2H-Ar), 7.98 (dd, 1H, *J* = 2.0 and 8.8 Hz, H-Ar), 8.05 (d, 2H, *J* = 8.8 Hz, H-Ar), 8.18 (d, 1H, *J* = 9.6 Hz, H4 of coumarin), 8.33 (d, 1H, *J* = 2.0 Hz, H5 of coumarin), 11.06 (s, 1H, NH), ^13^C NMR (101 MHz, DMSO-d_6_): δ = 118.41, 118.46, 118.64, 119.53, 123.03, 128.31, 129.40, 130.00, 130.79, 130.97, 133.12, 134.12, 135.49, 135.60, 142.51, 142.56, 143.79, 156.52(C9 of Coumarin), 159.50(C2 of Coumarin), 187.91(C = O). Anal. Calcd for C_24_H_16_ClNO_5_S (465.04) C, 61.87; H, 3.46; N, 3.01; Found: C, 62.05; H, 3.61; N, 3.18.

N-(4-(3-(4-bromophenyl)acryloyl)phenyl)-2-oxo-2H-chromene-6-sulfonamide **(5h)**

Pale yellow powder; yield 38%, m.p. 233–235°C. ^1^H NMR (400 MHz, DMSO-d_6_): δ = 6.59 (d, 1H, *J* = 9.6 Hz, H3 of coumarin), 7.26 (d, 2H, *J* = 8.8 Hz, H-Ar), 7.46–7.50 (m, 1H, H-Ar), 7.55–7.67 (m, 3H, CH = and 2H-Ar), 7.78–7.89 (m, 3H, = CH and 2H-Ar), 7.97 (dd, 1H, *J* = 2.0 and 8.4 Hz, H-Ar), 8.05 (d, 2H, *J* = 8.8 Hz, H-Ar), 8.17 (d, 1H, *J* = 9.6 Hz, H4 of coumarin), 8.32 (d, 1H, *J* = 2.0 Hz, H5 of coumarin), 11.05 (s, 1H, NH), ^13^C NMR (101 MHz, DMSO-d_6_): δ = 118.39, 118.45, 118.63, 119.52, 123.07, 124.36, 128.31, 129.38, 129.99, 130.32, 130.78, 130.95, 131.16, 132.31, 133.11, 134.11, 134.41, 135.60, 142.51, 143.78, 149.20, 156.47 (C9 of Coumarin), 159.49 (C2 of Coumarin), 187.90 (C = O). Anal. Calcd for C_24_H_16_BrNO_5_S (508.99) C, 56.48; H, 3.16; N, 2.74; Found: C, 56.66; H, 3.33; N, 2.92.

N-(4-(3-(4-nitrophenyl)acryloyl)phenyl)-2-oxo-2*H*-chromene-6-sulfonamide **(5i).**

Yellow powder; yield 52%, m.p. 274–275°C. ^1^H NMR (400 MHz, DMSO-d_6_): δ = 6.60 (d, 1H, *J* = 9.6 Hz, H3 of coumarin), 7.28 (d, 2H, *J* = 8.8 Hz, H-Ar), 7.58(d, 1H, *J* = 8.8 Hz, H-Ar), 7.73 (d, 1H, *J* = 15.6 Hz, CH =), 7.98 (dd, 1H, *J* = 2.4 and 8.8 Hz, H-Ar), 8.02 (d, 1H, *J* = 16.0 Hz, = CH), 8.08–8.13(m, 4H, H-Ar), 8.18 (d, 1H, *J* = 9.6 Hz, H4 of coumarin), 8.26 (d, 1H, *J* = 8.8 Hz, 2H-Ar-NO_2_), 8.34 (d, 1H, *J* = 2.4 Hz, H5 of coumarin), 11.10 (s, 1H, NH), ^13^C NMR (101 MHz, DMSO-d_6_): δ = 118.41, 118.47, 118.60, 119.54, 124.39, 126.35, 128.32, 130.01, 130.24, 130.97, 132.79, 135.61, 141.15, 141.69, 142.81, 143.79, 148.49, 156.57 (C9 of Coumarin), 159.50 (C2 of Coumarin), 187.82 (C = O). Anal. Calcd for C_24_H_16_N_2_O_7_S (476.07) C, 60.50; H, 3.38; N, 5.88; Found: C, 60.66; H, 3.52; N, 6.01.

### 4.2. Enzymology

#### 4.2.1. Materials

Reduced GSH, 1-chloro-2,4-dinitrobenzene (CDNB), ampicillin, sodium dodecyl sulfate (SDS) and the chromatographic material Sepharose CL-6B were purchased from Sigma-Aldrich, USA (Merck) and were used without further treatment. Ethanol, methanol, and dimethyl sulfoxide (DMSO) were purchased from Scharlau (Spain).

#### 4.2.2. Expression and purification of hGSTP1-1 from recombinant *E*. *coli* cells

The expression and purification of the hGSTP1-1 were based on a published method [34]. The purity of the enzyme was evaluated by sodium dodecyl sulphate polyacrylamide gel electrophoresis (SDS-PAGE). Purified enzyme fractions were pooled, diluted by dropwise addition of glycerol (to 50% v/v final concentration) and stored at -20°C.

#### 4.2.3. Protein determination

Protein concentration was determined according to Bradford assay using bovine serum albumin as a standard [68].

#### 4.2.4. Enzyme assays and inhibition analysis

Determination of hGSTP1-1 activity was carried out in potassium phosphate buffer (100 mM, pH 6.5, 1 mL total volume), at 25°C using as substrates CDNB and GSH. The reaction was monitoring at 340 nm for 120 s, as described previously [34]. For inhibition analysis, coumarin derivatives were dissolved in DMSO (100 μΜ) and added to the assay mixture at 10 μΜ final concentration. The mixture was incubated at 25°C for 1 min, prior adding the enzyme sample. The IC_50_ values were determined from a graph of the remaining GST activity (%) against inhibitor concentration. GraphPad Prism 9.3.1 (GraphPad Prism Software, Inc.) was used for determination of IC_50_ values. Kinetic inhibition analysis was performed as previously described [34] with minor modifications. Initial velocities using CDNB as a variable substrate (typically 14–1000 μM) were determined in the presence of constant concentration of GSH (2.5 mM) in the absence and presence of the coumarin derivative **5g** (0–50 μM). Initial velocities using GSH as a variable substrate (typically 37.5–3750 μΜ) were determined in the presence of 1 mM CDNB in the absence and presence of the coumarin derivative 5g (0–50 μM). Enzyme assays were performed in triplicates and initial velocities were corrected for spontaneous reaction rates.

#### 4.2.5. Cytotoxicity studies of the most potent inhibitors against PC3, DU-145 and MCF-7 cancer cell lines

The MTT colorimetric assay was used to evaluate the *in vitro* cytotoxicity of **5a, 5f** and **5g** coumarin derivatives against PC3, DU-145 and MCF-7 cancer cell lines. Cancer cells were inoculated into a 96-well plate (initially at a density of 8 x 10^3^ cells/well in 100 μL of culture medium) and were cultivated for 24 h, at 37°C in a CO_2_ incubator (5%). Different concentrations of 5a, 5f and 5g derivatives (1.5 μΜ to 200 μΜ), were added to cultures and incubated for 48 h. In all experiments the final DMSO concentration did not exceed 0.2% (v/v) in culture medium. The culture medium was then replaced with 100 μL of MTT solution (1 mg/mL) and after 2 h incubation, the solution was aspirated and the produced formazan crystals were dissolved in isopropanol (100 μL). The absorbance was measured at 540 nm. Dose–cell viability graphs were created using GraphPad Prism 9.3.1 and the CC_50_ values were determined.

### 4.3. Computational studies

#### 4.3.1. Molecular docking

The molecular docking protocol based on the CANDOCK algorithm [64] was carried out to generate the starting models of the three most potent coumarin derivatives **5a**, **5f**, and **5g** at the active site of glutathione transferase 1–1 (hGSTP1-1). The obtained poses were evaluated by the radial-mean-reduced scoring function at a cutoff radius of 6 Å from each atom of the ligand (RMR6). The X-ray crystal structure of hGSTP1-1 (PDB ID: 18GS, chain A) was obtained from the protein data bank. The 3D structures of three studied coumarin derivatives were prepared in Avogadro [69] and subsequently geometrically optimized with the Hartree-Fock method and 6-31G(d) basis set using Gaussian 16 program [70]. As the crystal structures of **5a**, **5f**, and **5g** in complex with hGSTP1-1 have not been experimentally determined yet, the binding modes of the studied derivatives with the lowest docking score values were selected.

## Supporting information

S1 File (PDF)
