# Supplementary figures and images for: Novel coumarin-6-sulfonamide-chalcone hybrids as glutathione transferase P1-1 inhibitors
Source: PLoS One. 2024 Aug 14;19(8):e0306124. doi: 10.1371/journal.pone.0306124 (PMC11324126; doi:10.1371/journal.pone.0306124)

## Supporting information

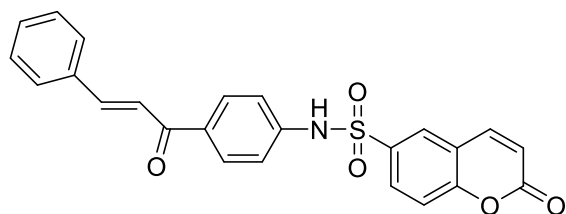

**5a**

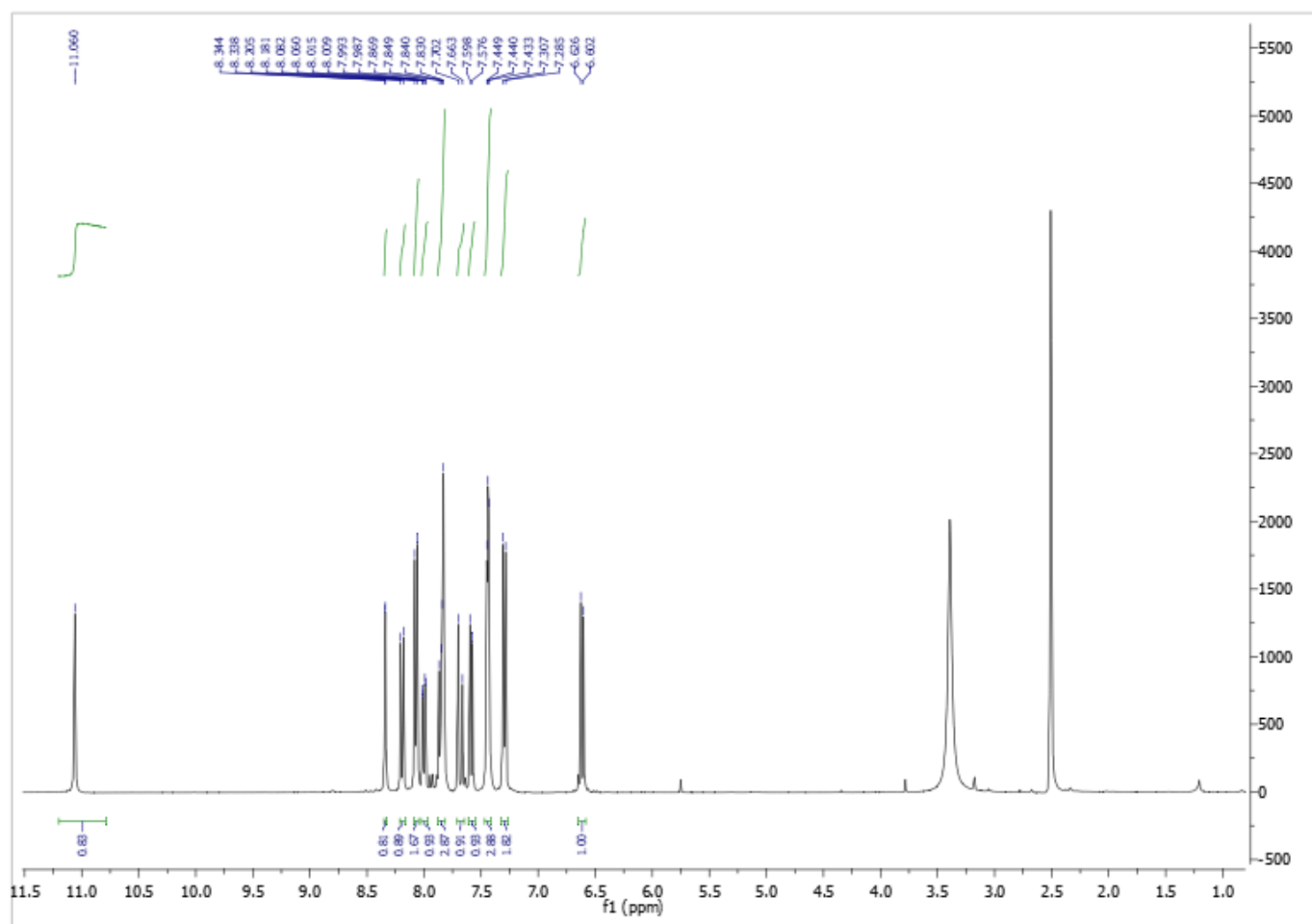

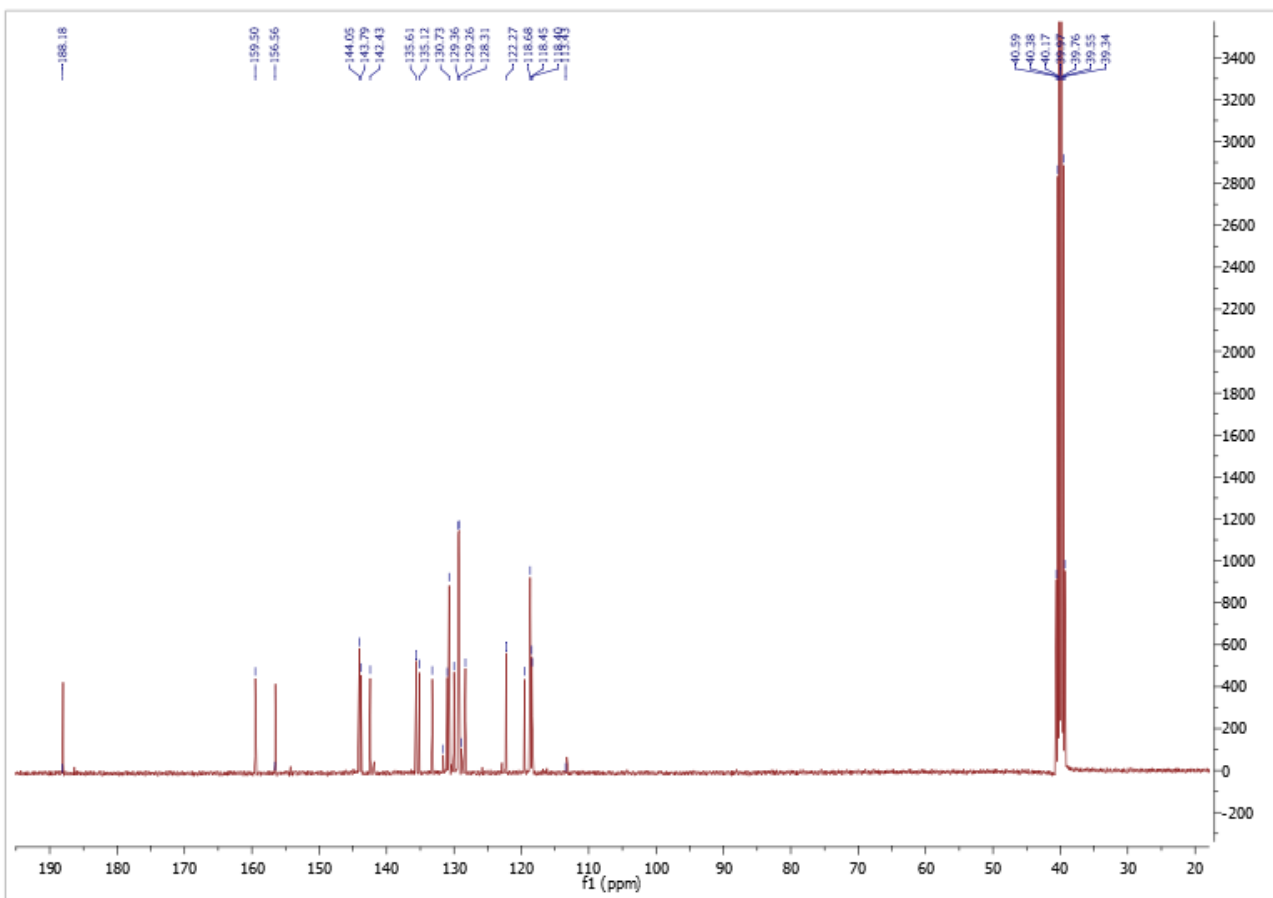

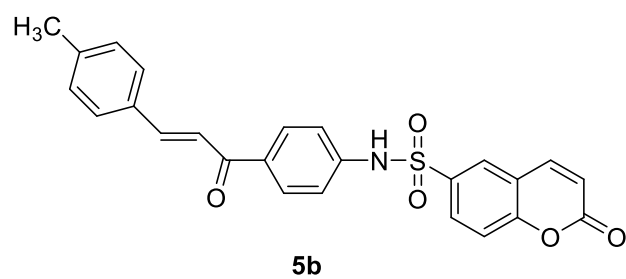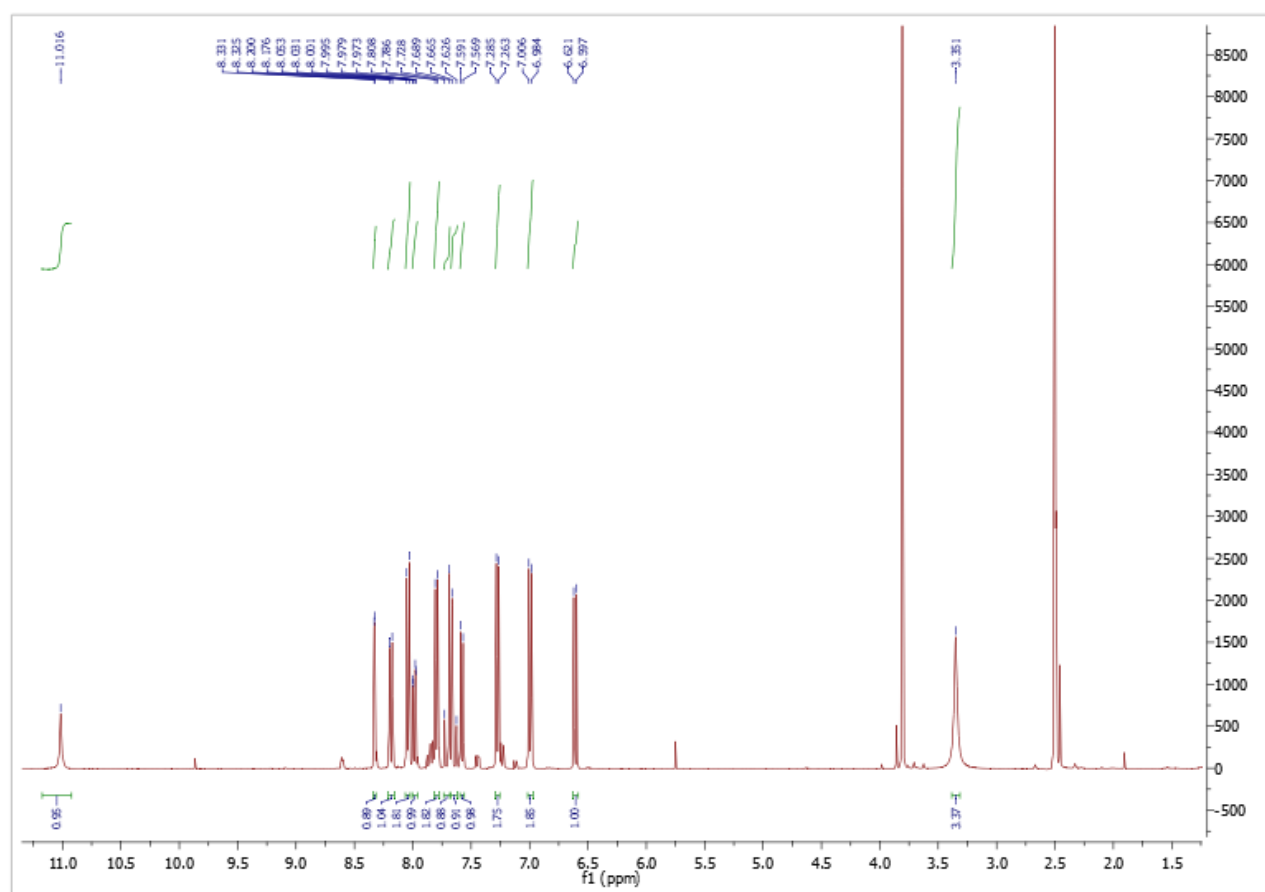

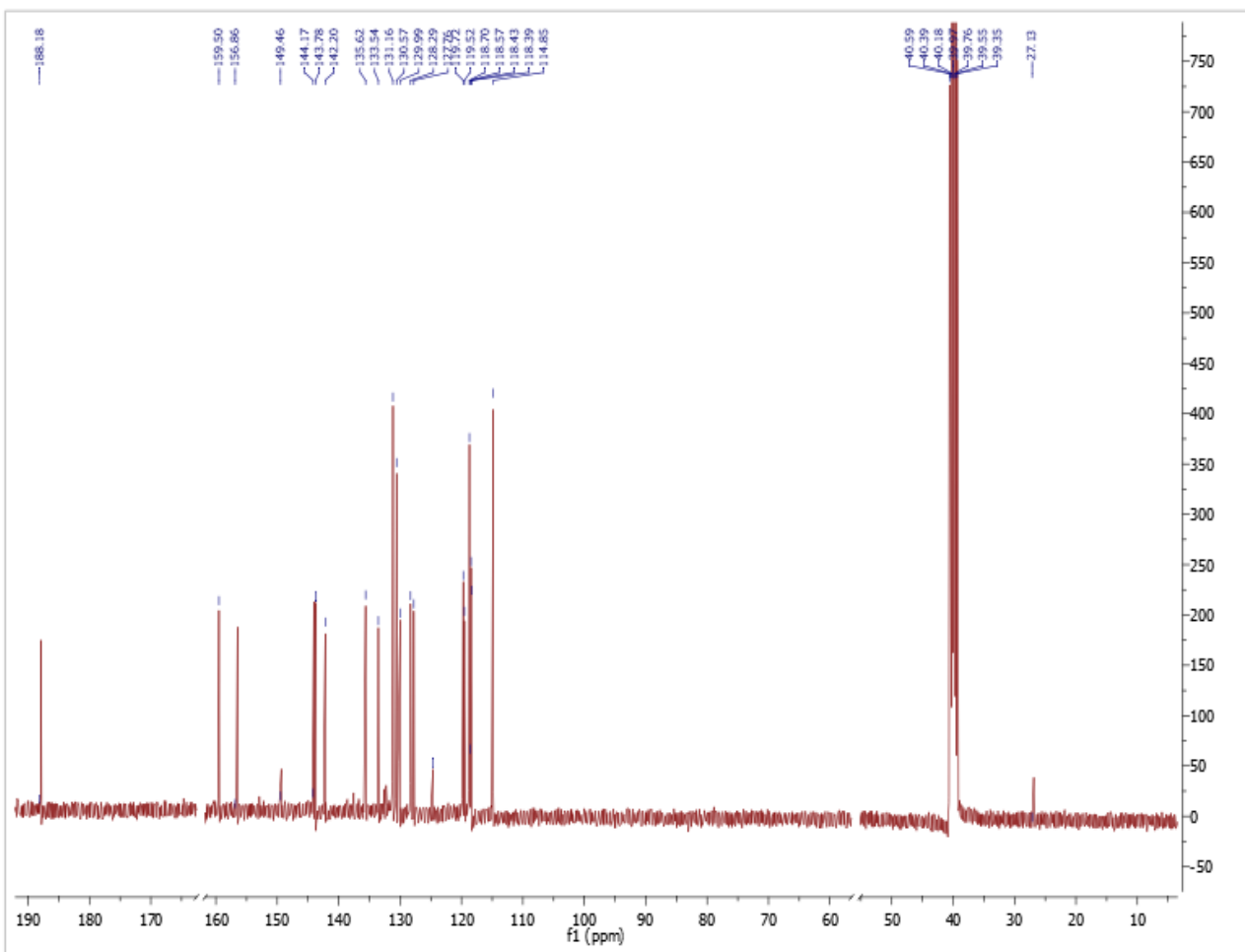

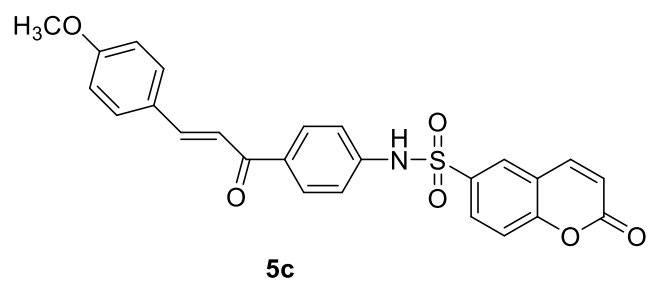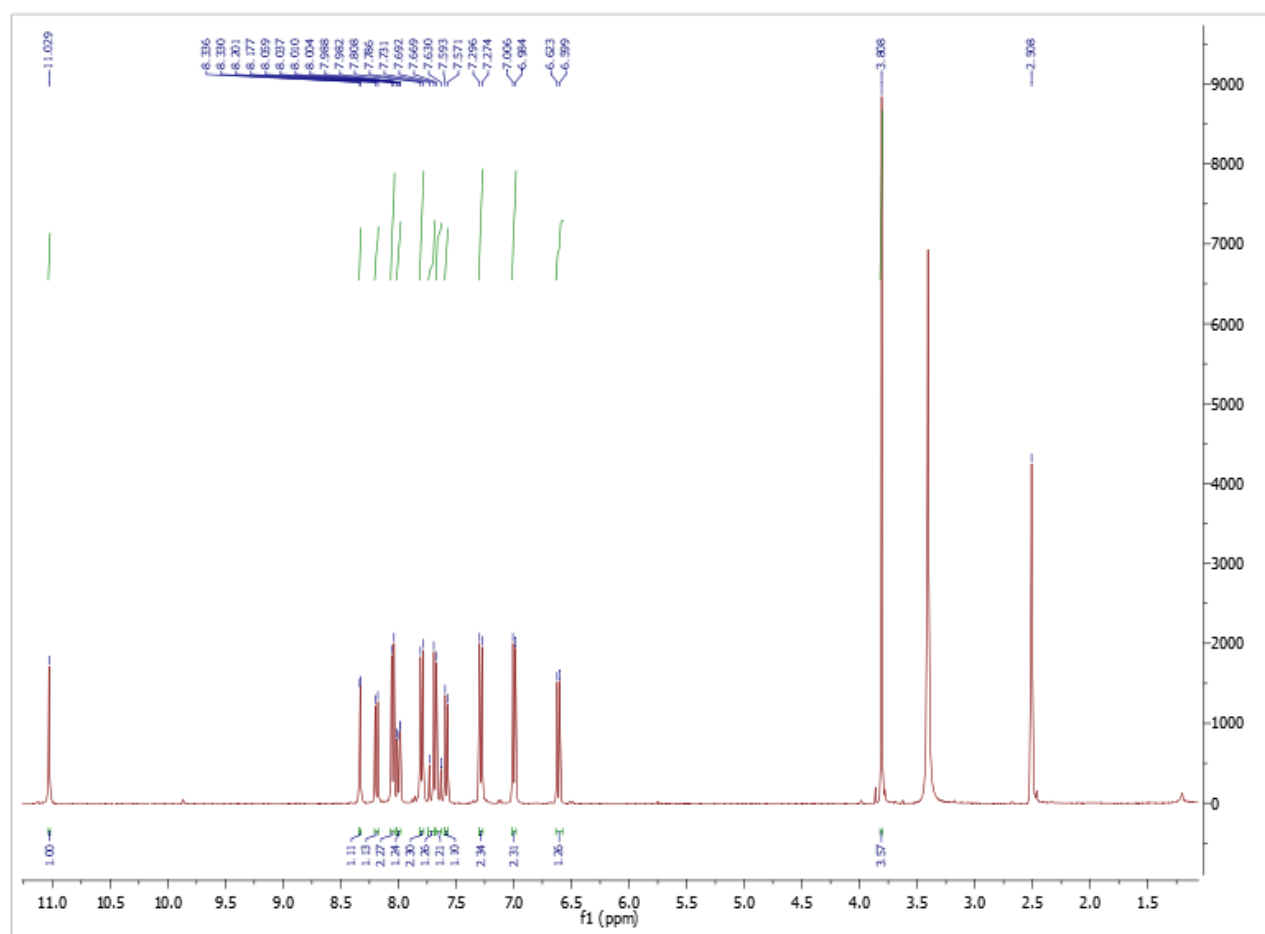

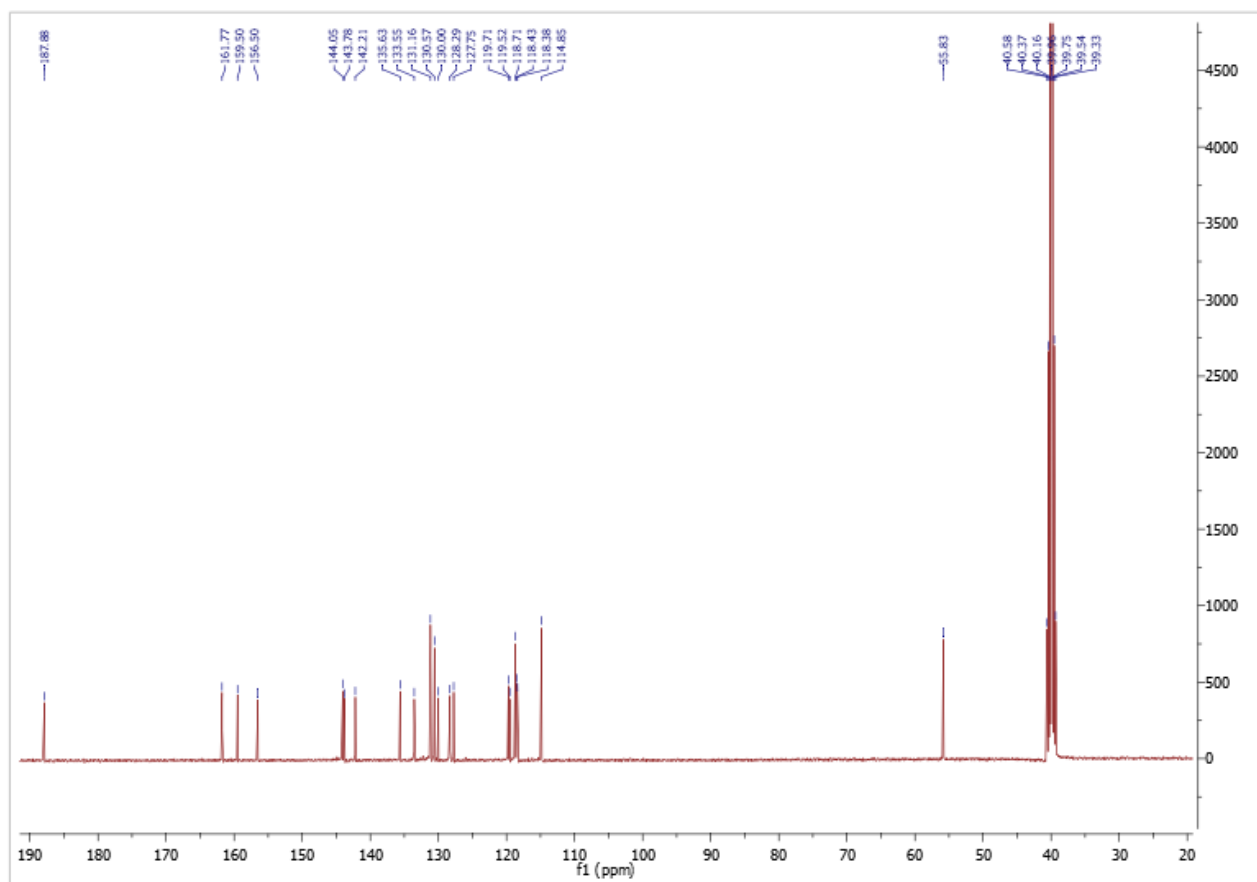

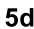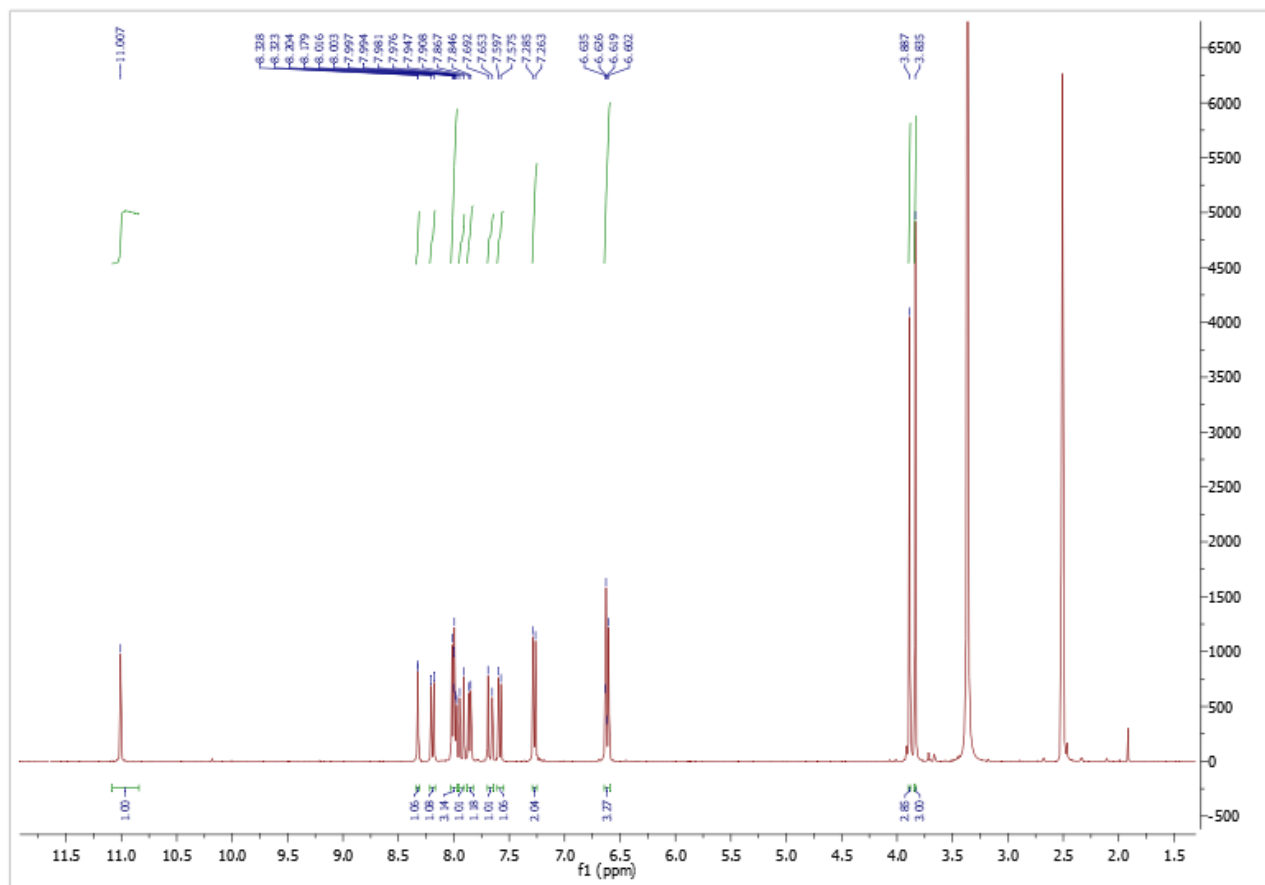

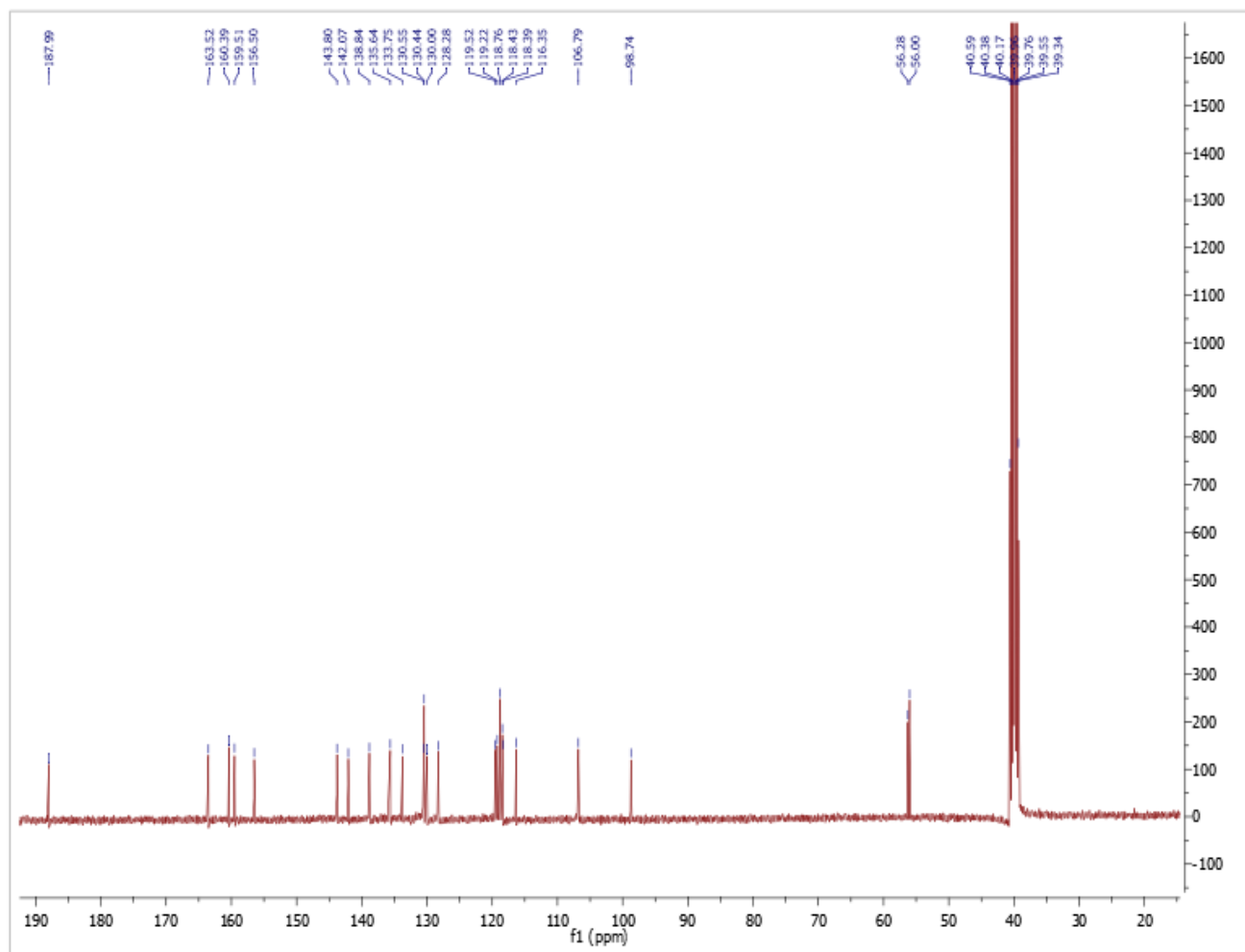

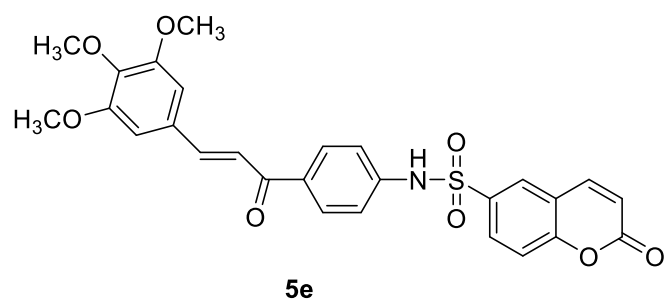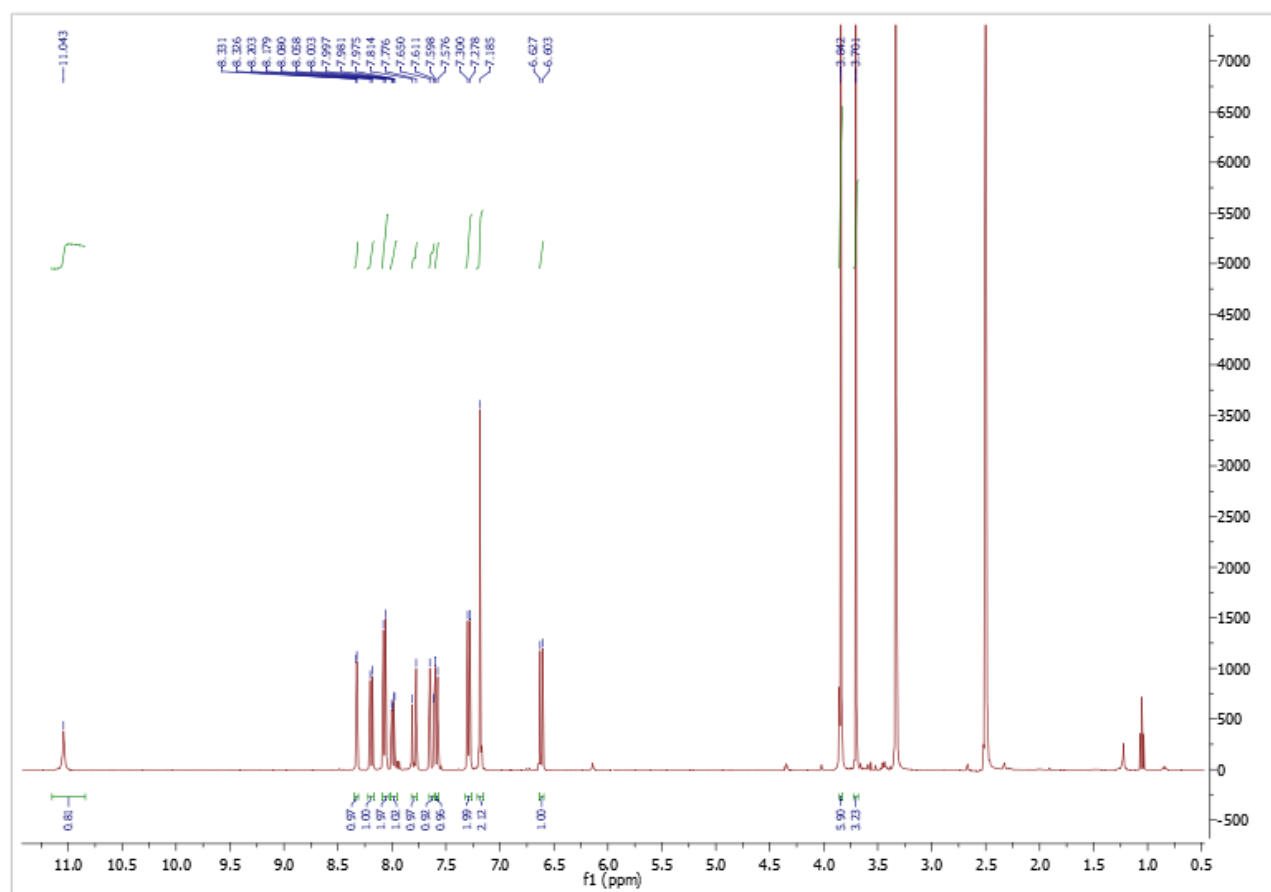

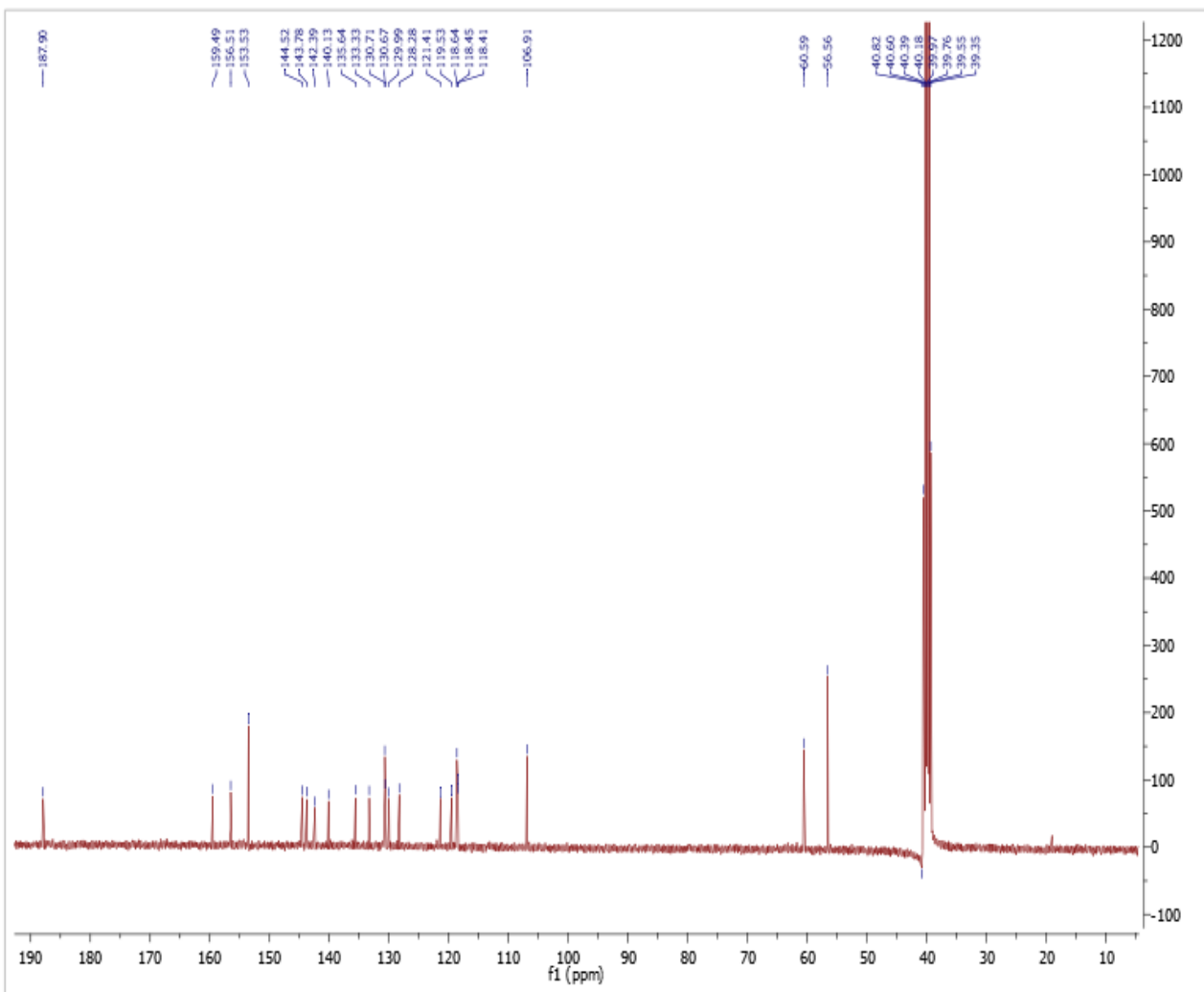

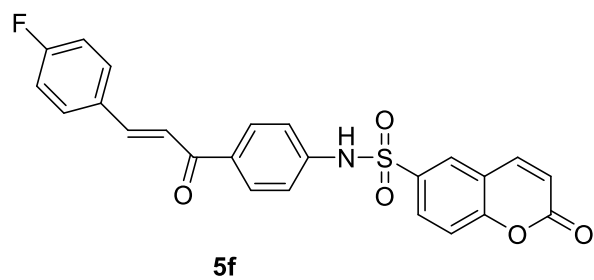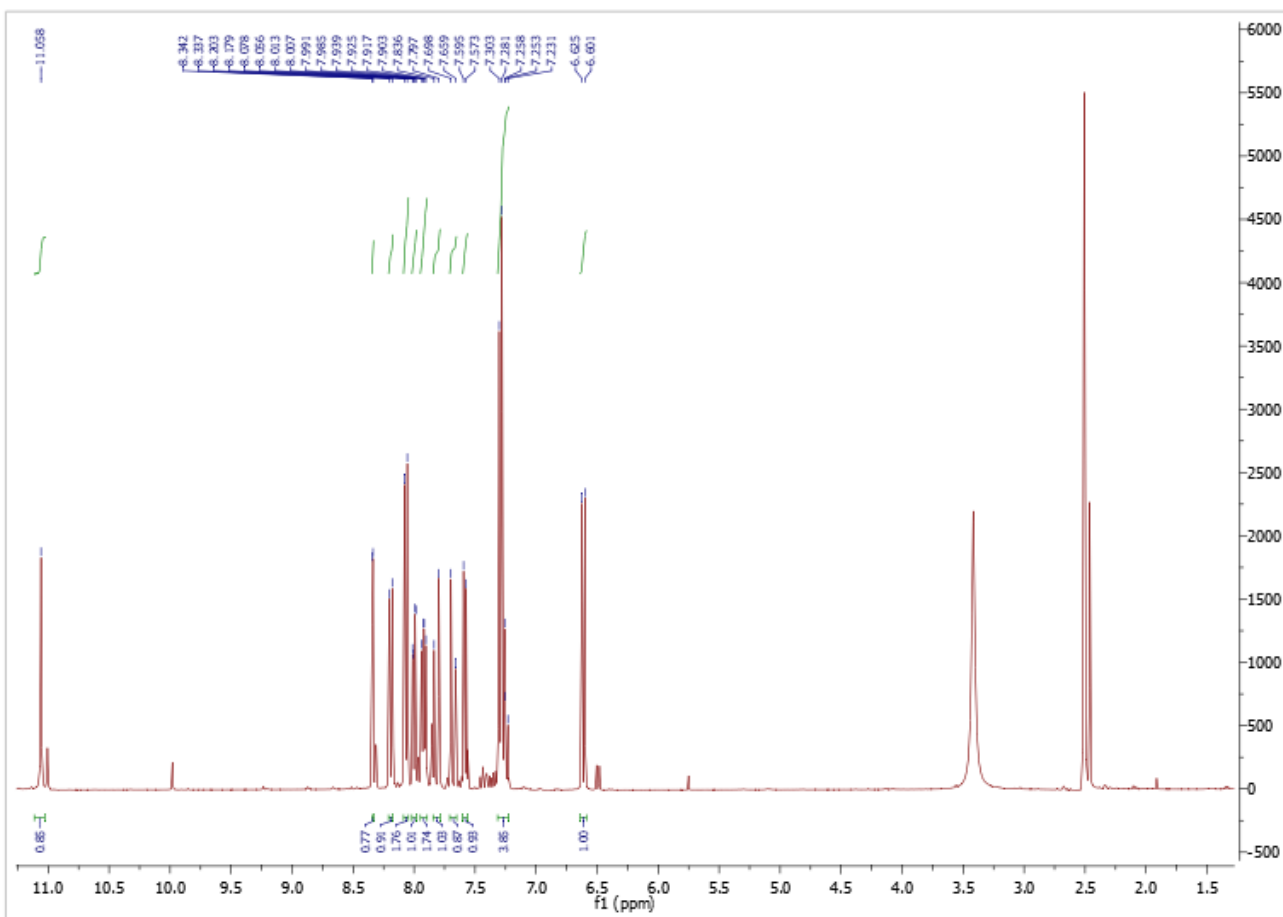

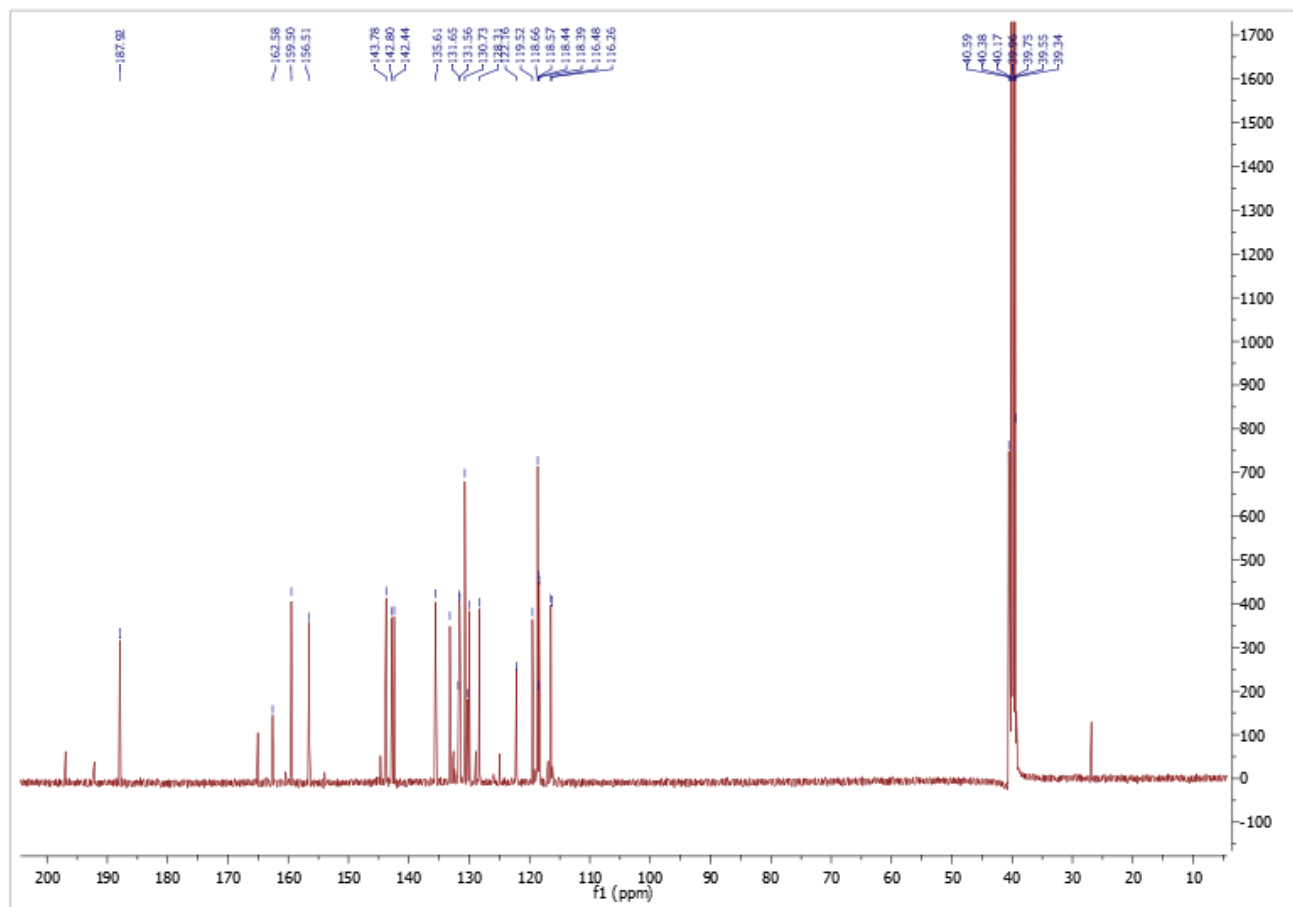

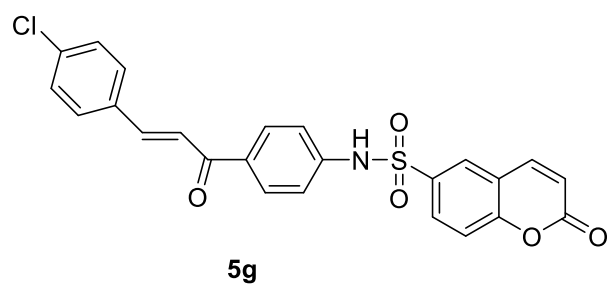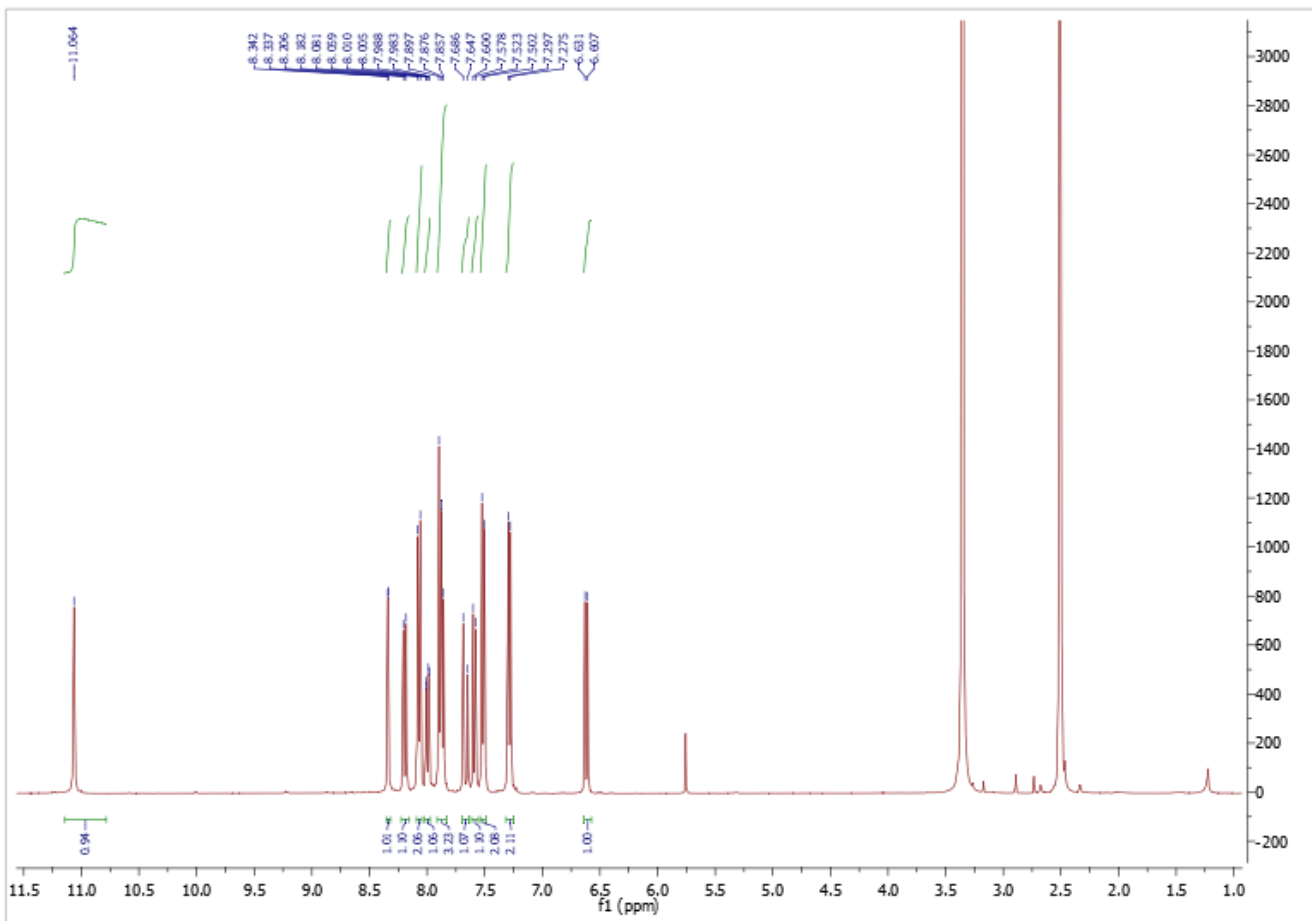

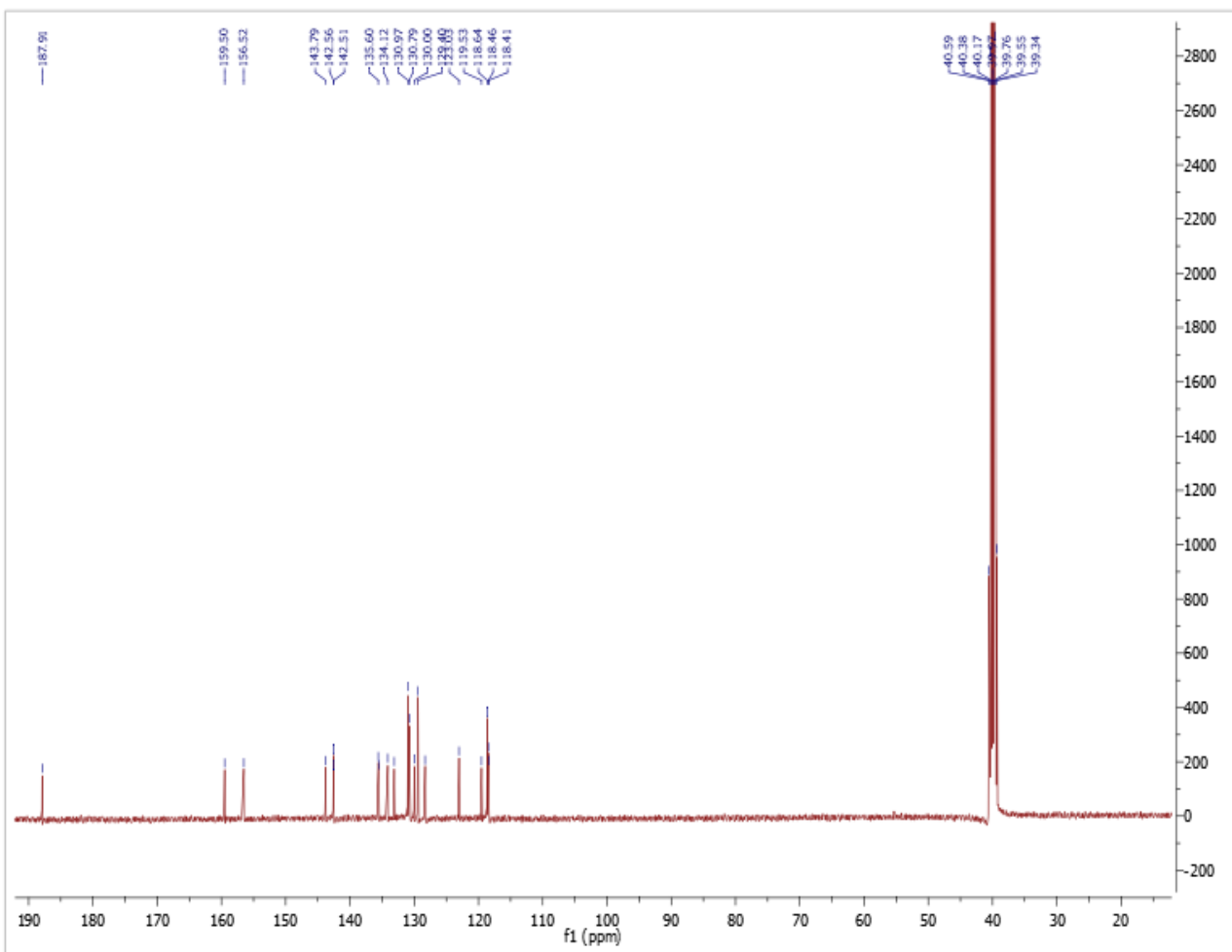

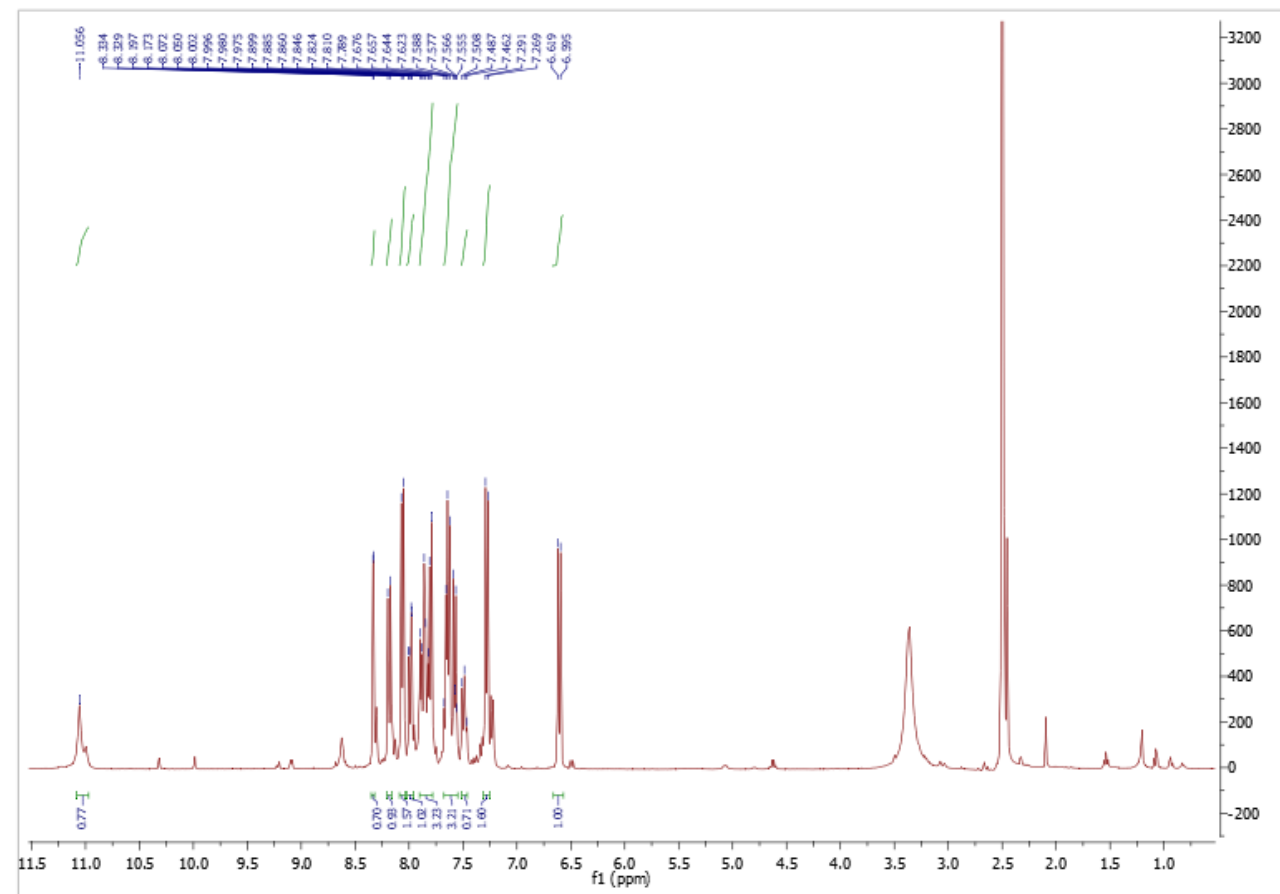

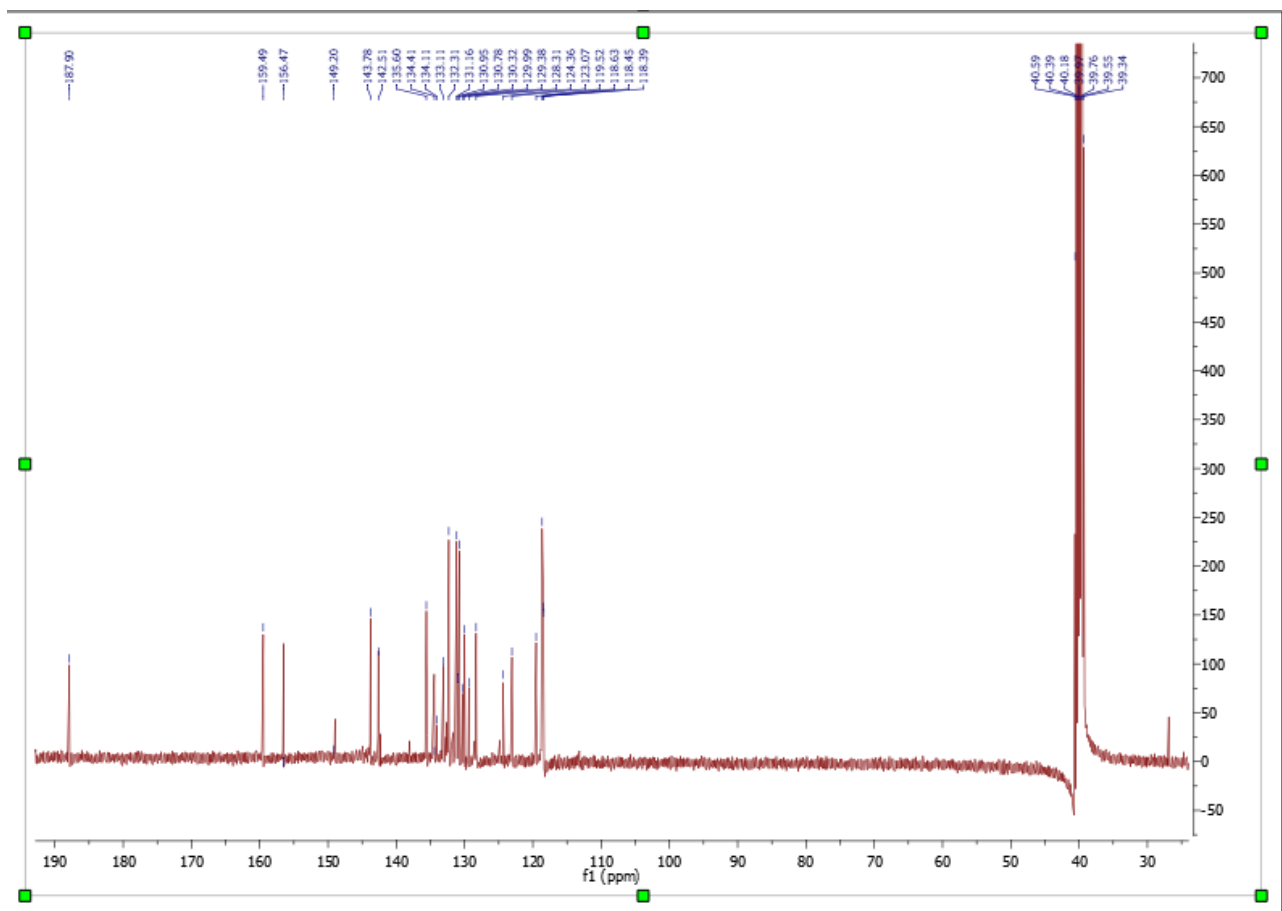

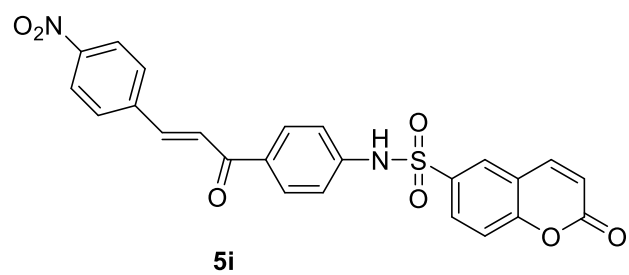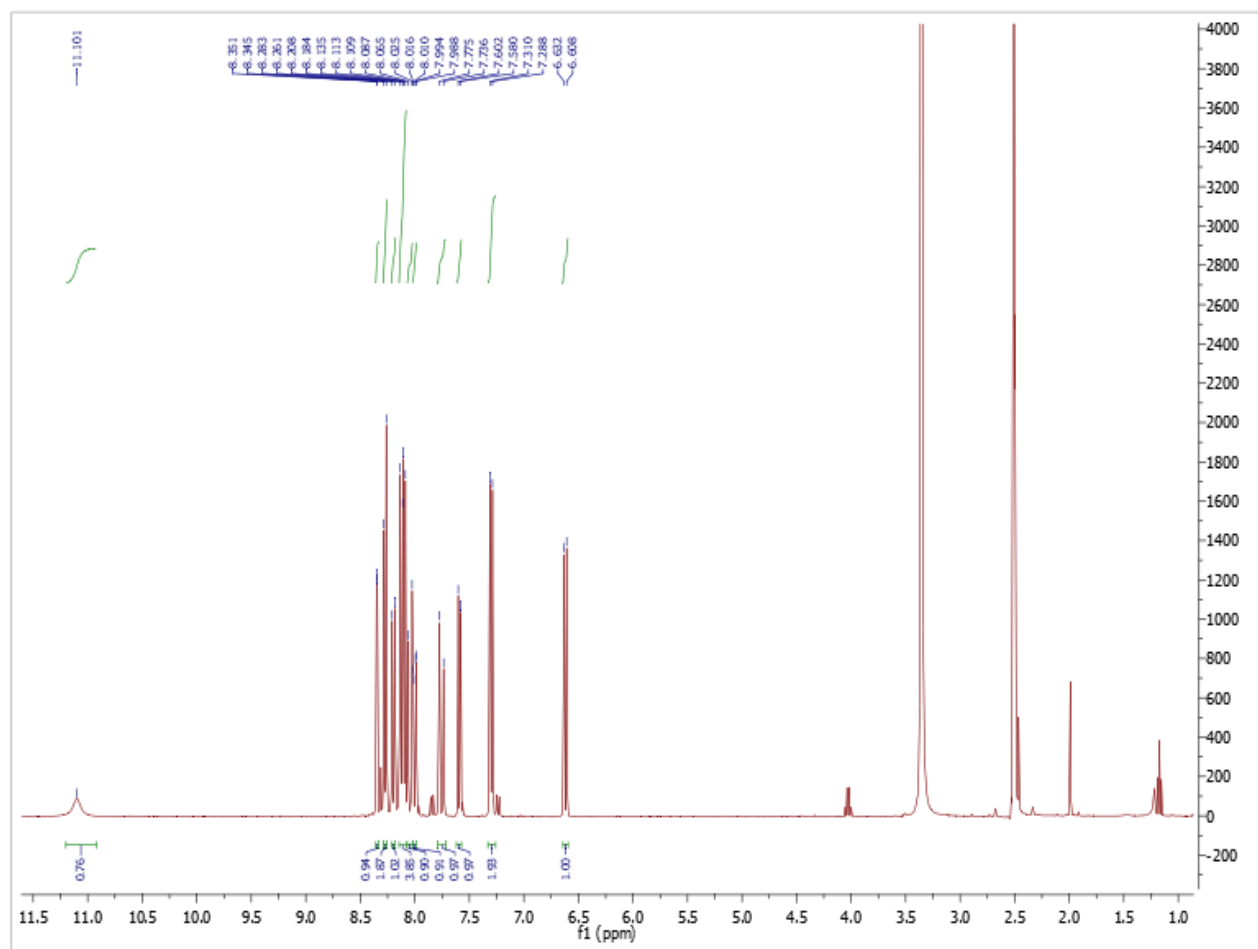

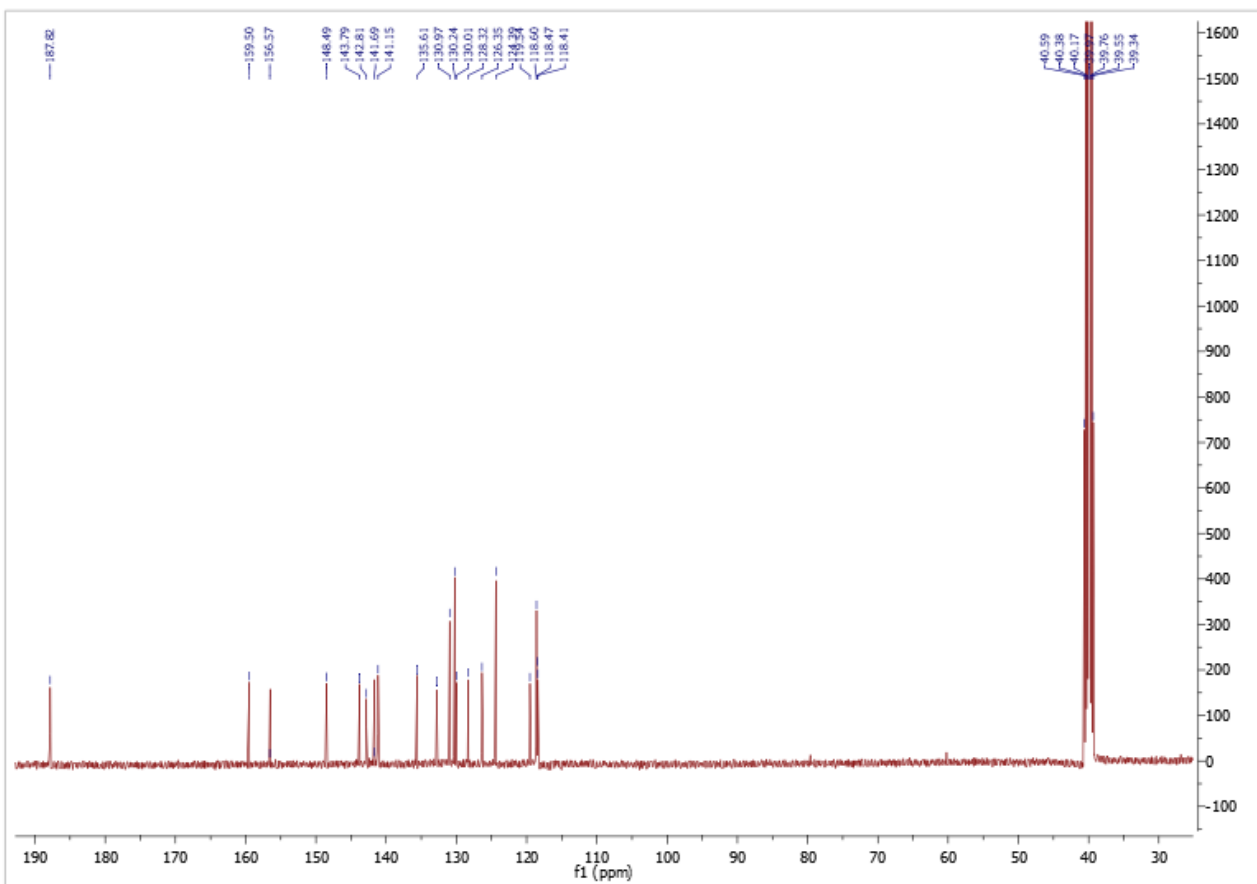

Supplement: S1 File — (PDF) [file pone.0306124.s001.pdf]
